# Using interpretable machine learning to extend heterogeneous antibody-virus datasets

**DOI:** 10.1016/j.crmeth.2023.100540

**Published:** 2023-07-25

**Authors:** Tal Einav, Rong Ma

**Affiliations:** 1 Basic Sciences Division and Computational Biology Program, Fred Hutchinson Cancer Research Center, Seattle, WA 98109, USA; 2 Center for Infectious Disease and Vaccine Research, La Jolla Institute for Immunology, La Jolla, CA 92037, USA; 3 Department of Statistics, Stanford University, Stanford, CA 94305, USA

**Keywords:** antibody-virus interactions, influenza, matrix completion, imputation, error estimation, serology, hemagglutination inhibition

## Abstract

A central challenge in biology is to use existing measurements to predict the outcomes of future experiments. For the rapidly evolving influenza virus, variants examined in one study will often have little to no overlap with other studies, making it difficult to discern patterns or unify datasets. We develop a computational framework that predicts how an antibody or serum would inhibit any variant from *any other study*. We validate this method using hemagglutination inhibition data from seven studies and predict 2,000,000 new values ± uncertainties. Our analysis quantifies the transferability between vaccination and infection studies in humans and ferrets, shows that serum potency is negatively correlated with breadth, and provides a tool for pandemic preparedness. In essence, this approach enables a shift in perspective when analyzing data from “what you see is what you get” into “what anyone sees is what everyone gets.”

## Highlights

• Predict unmeasured antibody-virus interactions across multiple studies
• Empower direct comparisons between studies through expanded virus panels
• Determine which criteria (age, exposure history) lead to distinct responses
• Assess how accurately animal data predict the human antibody response

## Motivation

To quantify the immune response against a rapidly evolving virus, groups routinely measure antibody inhibition against many virus variants. Over time, the variants being studied change, and there is a need for methods that infer missing interactions and distinguish between confident predictions and hallucinations. Here, we develop a matrix completion framework that uses patterns in antibody-virus inhibition to infer the value and confidence of unmeasured interactions. This same approach can combine general datasets—from drug-cell interactions to user movie preferences—that have partially overlapping features.

## Graphical abstract

**Figure:**
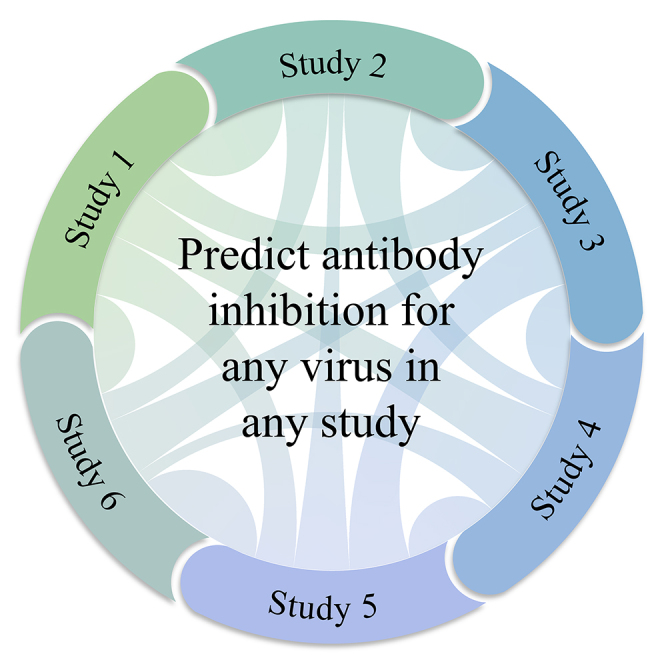

## Teaser

Einav and Ma develop a framework to infer unmeasured antibody-virus interactions. If three studies measure antibody inhibition against viruses 1–30, 10–40, and 20–50, respectively, then their approach predicts how any antibody inhibits all 50 viruses. As more datasets are combined, the number of predictions rapidly increases, and prediction accuracy improves.

## Introduction

Our understanding of how antibody-mediated immunity drives viral evolution and escape relies upon painstaking measurements of antibody binding, inhibition, or neutralization against variants of concern.^1^ While antibodies can cross-react and inhibit multiple variants, viral evolution slowly degrades such immunity, leading to periodic reinfections that elicit new antibodies. To get an accurate snapshot of this complex response, we must not only measure inhibition against currently circulating strains but also against historical variants.^2^^,^^3^

**Figure 1:**
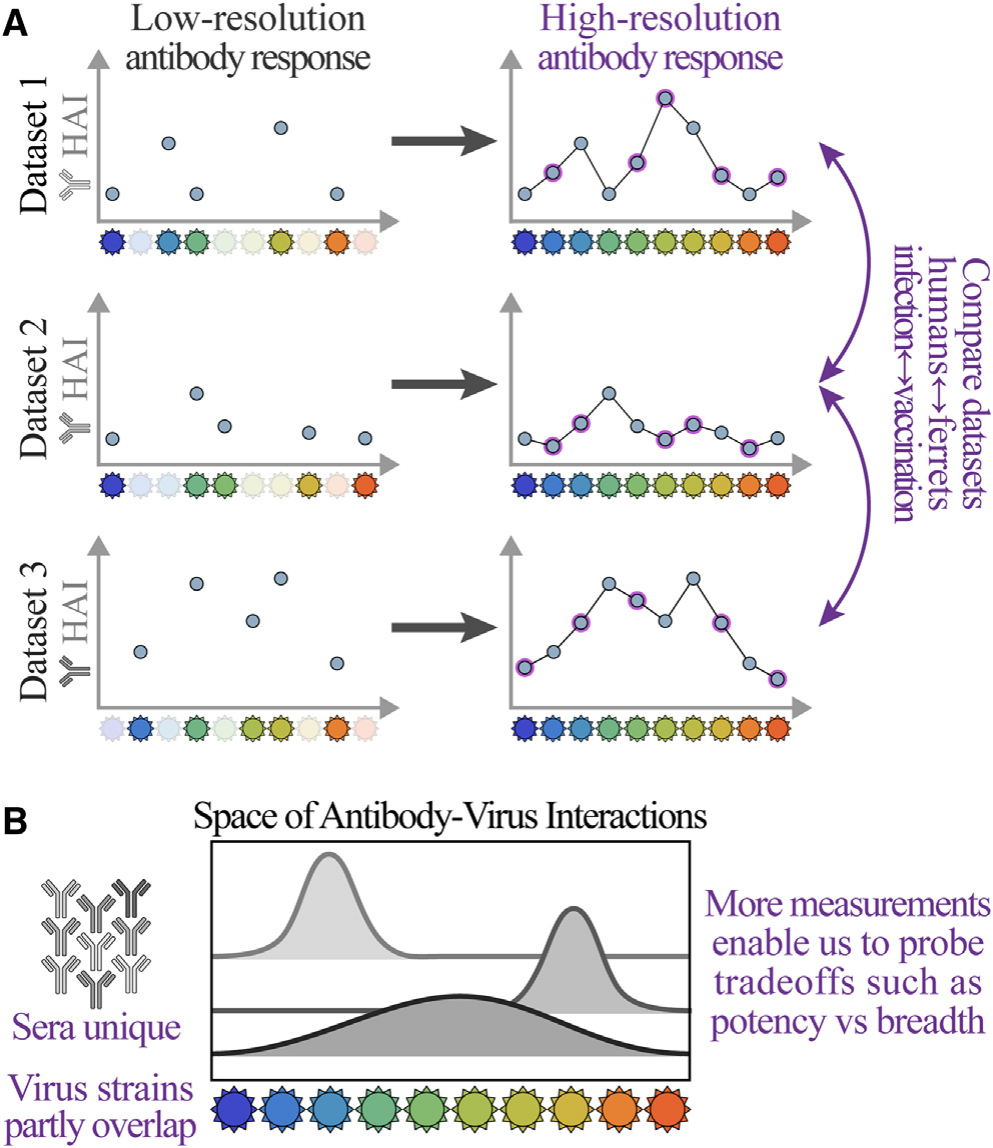
Challenges of comparing antibody-virus datasets (A) We develop a framework that predicts antibody responses (e.g., binding, hemagglutination inhibition [HAI], or neutralization) of any serum against viral variants from any other dataset, enabling direct cross-study comparison. (B) Because each serum is unique and virus panels often only partially overlap, these expanded measurements are necessary to characterize the limits of the antibody response or quantify tradeoffs between key features, such as potency (the strength of a response) vs. breadth (how many viruses are inhibited).

Every antibody-virus interaction is unique because (1) the antibody response (serum) changes even in the absence of viral exposure and (2) for rapidly evolving viruses such as influenza, the specific variants examined in one study will often have little to no overlap with other studies (Figure 1). This lack of crosstalk hampers our ability to comprehensively characterize viral antigenicity, predict the outcomes of viral evolution, and determine the best composition for the annual influenza vaccine.^4^

**Figure 2:**
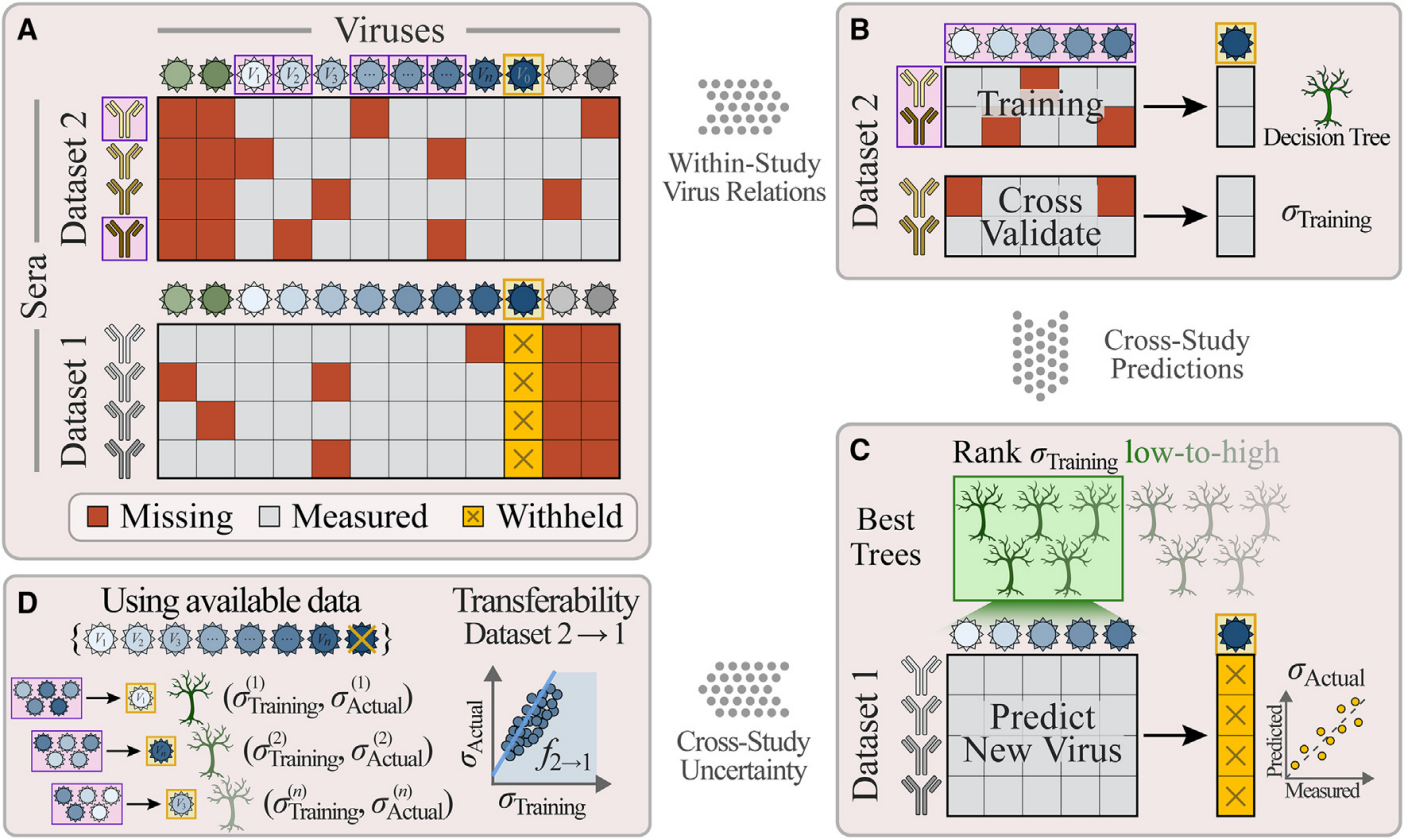
Combining datasets to predict values and uncertainties for missing viruses (A) Schematic of data availability; two studies measure antibody responses against overlapping viruses (shades of blue) as well as unique viruses (green/gray). Studies may have different fractions of missing values (dark-red boxes) and measured values (gray). To test whether virus behavior can be inferred across studies, we predict the titers of a virus in dataset 1 (*V*_0_, gold squares), using measurements from the overlapping viruses (*V*_1_–*V*_*n*_) as features in a random forest model. (B) We train a decision tree model using a random subset of antibodies and viruses from dataset 2 (boxed in purple), cross-validate against the remaining antibody responses in dataset 2, and compute the root-mean-square error (RMSE, denoted by *σ*_Training_). (C) Multiple decision trees are trained, and the average from the 5 trees with the lowest error are used as the model going forward. Applying this model to dataset 1 (which was not used during training) yields the desired predictions, whose RMSE is given by *σ*_Actual_. We repeat this process, withholding each virus in every dataset. (D) To estimate the prediction error *σ*_Actual_ (which we are not allowed to directly compute because *V*_0_’s titers are withheld), we define the transferability relation *f*_2→1_ between the training error *σ*_Training_ in dataset 2 and actual error *σ*_Actual_ in dataset 1 using the decision trees that predict viruses *V*_1_–*V*_*n*_ (without using *V*_0_). Applying this relation to the training error, *f*_2→1_(*σ*_Training_), estimates *σ*_Actual_ for *V*_0_.

In this work, we develop a new cross-study matrix completion algorithm that leverages patterns in antibody-virus inhibition data to infer unmeasured interactions. Specifically, we demonstrate that multiple datasets can be combined to predict the behavior of viruses that were entirely absent from one or more datasets (e.g., Figure 2A, predicting values for the green viruses in dataset 2 and the gray viruses in dataset 1). Whereas past efforts could only predict values for partially observed viruses within a single dataset (i.e., predicting the red squares for the blue/gray viruses in dataset 2 or the green/blue viruses in dataset 1),^5^^,^^6^^,^^7^ here we predict the behavior of viruses that do not have a single measurement in a dataset.

Algorithms that predict the behavior of large virus panels are crucial because they render the immunological landscape in higher resolution, helping to reveal which viruses are potently inhibited and which escape antibody immunity.^3^^,^^4^ For example, polyclonal human sera that strongly neutralize one virus may exhibit 10× weaker neutralization against a variant with one additional mutation.^8^ Given the immense diversity and rapid evolution of viruses, it behooves us to pool together measurements from different studies and build a more comprehensive description of serum behavior.

Even when each dataset is individually complete, many interactions can still be inferred by combining studies. The seven datasets examined in this work measured 60%–100% of interactions between their specific virus panel and sera, but against an expanded virus panel containing all variants, fewer than 10% of interactions were measured. Moreover, the missing entries are highly structured, with entire columns (representing viruses; Figure 2A) missing from each dataset. This introduces unique challenges because most matrix completion or imputation methods require missing entries to be randomly distributed,^5^^,^^9^^,^^10^^,^^11^^,^^12^^,^^13^ and the few methods tailored for structured missing data focus on special classes of generative models that are less effective in this context.^14^^,^^15^^,^^16^ In contrast, we construct a framework that harnesses the specific structure of these missing values, enabling us to predict over 2,000,000 new values comprising the remaining 90% of interactions.

The key feature we develop that enables matrix completion across studies is error quantification. Despite numerous algorithms to infer missing values, only a few methods exist that can estimate the error of these predictions under the assumption that missing values are randomly distributed,^17^^,^^18^ and to our knowledge, no methods can quantify error for general patterns of missing data. Because we do not know *a priori* whether datasets can inform one another, it is crucial to estimate the confidence of cross-study predictions. Our framework does so using a data-driven approach to quantify the *individual error* of each prediction so that users can focus on high-confidence inferences (e.g., those with ≤4-fold error) or search for additional datasets that would further reduce this uncertainty.

Our results provide guiding principles in data acquisition and promote the discovery of new mechanisms in several key ways: (1) Existing antibody-virus datasets can be unified to predict each serum against any virus, providing a massive expansion of data and fine-grained resolution of these antibody responses. (2) This expanded virus panel enables an unprecedented direct comparison of human ↔ ferret and vaccination ↔ infection studies, quantifying how distinct the antibody responses are in each category. (3) Using the expanded data, we explore the relation between two key features of the antibody response, showing the tradeoff between potency and breadth. (4) We demonstrate an application for pandemic preparedness, where the inhibition of a new variant measured in one study is immediately extrapolated to other datasets. (5) Our approach paves the way to rationally design virus panels in future studies, saving time and resources by measuring a substantially smaller set of viruses. In particular, we determine which viruses will be maximally informative and quantify the benefits of measuring each additional virus.

Although this work focuses on antibody-virus inhibition measurements for influenza, it readily generalizes to other viruses, other assays (e.g., using binding or neutralization), and more general applications involving intrinsically low-dimensional datasets.

## Results

### The low dimensionality of antibody-virus interactions empowers matrix completion

Given the vast diversity of antibodies, it is easy to imagine that serum responses cannot inform one another. Indeed, many factors, including age, geographic location, frequency/type of vaccinations, and infection history, shape the antibody repertoire and influence how it responds to a vaccine or a new viral threat.^19^^,^^20^^,^^21^^,^^22^^,^^23^

Yet much of the heterogeneity of antibody responses found through sequencing^24^ collapses when we consider functional behavior such as binding, inhibition, or neutralization against viruses.^25^^,^^26^ Previous work has shown that antibody-virus inhibition data are intrinsically low dimensional,^27^ which spurred applications ranging from antigenic maps to the recovery of missing values from partially observed data.^5^^,^^6^^,^^7^^,^^28^ However, these efforts have almost exclusively focused on individual datasets of ferret sera generated under controlled laboratory conditions, circumventing the many obstacles of predicting across heterogeneous human studies.

In the following sections, we develop a matrix completion algorithm that predicts measurements for a virus in dataset 1 (e.g., the virus-of-interest in Figure 2A, boxed in gold) by finding universal relationships between the other overlapping viruses and the virus-of-interest in dataset 2 and applying them to dataset 1. We first demonstrate the accuracy of matrix completion by withholding *all* hemagglutination inhibition (HAI) measurements from one virus in one dataset (Figure 2A, gold boxes) and using the other datasets to generate *predictions ± errors*, where each *error* quantifies the uncertainty of a *prediction*. Although we seek accurate predictions with low estimated error, it may be impossible to accurately predict some interactions (e.g., measurements of viruses from 2000–2010 may not be able to predict a distant virus from 1970), and those error estimates should be larger to faithfully reflect this uncertainty. After validating our approach on seven large serological studies, we apply matrix completion to greatly extend their measurements.

### Cross-study matrix completion using a random forest

We first predict virus behavior between two studies before considering multiple studies. Figure 2 and Box 1 summarize *leave-one-out* *analysis*, where a virus-of-interest *V*_0_ is withheld from one dataset (Figure 2A, blue virus boxed in gold). We create multiple decision trees using a subset of overlapping viruses *V*_1_, *V*_2_…*V*_*n*_ as features and a subset of antibody responses within dataset 2 for training (STAR Methods). These trees are cross-validated using the remaining antibody responses from dataset 2 to quantify each tree’s error *σ*_Training_, and we predict *V*_0_ in dataset 1 using the average of the values ± errors from the 5 best trees with the lowest error (Figures 2B and 2C; Box 1).

Box 1 **Predicting virus behavior (value ± error) across studies**

*Input:*

• Dataset-of-interest *D*_0_ containing virus-of-interest *V*_0_ whose measurements we predict
• Other datasets {*D*_*j*_}, each containing *V*_0_ and at least 5 viruses *V*_*j*,1_, *V*_*j*,2_… that overlap with the *D*_0_ virus panel, used to extrapolate virus behavior
• Antibody responses *A*_*j*,1_, *A*_*j*,2_… in each dataset *D*_*j*_. When *j* ≠ 0, we only consider antibody responses with non-missing values against *V*_0_

*Steps*:

1. For each *D*_*j*_, create *n*_Tree__s_ = 50 decision trees predicting *V*_0_ based on *n*_Features_ = 5 other viruses and a fraction *f*_Samples_ = 3/10 of sera
  ○ For robust training, we restrict attention to features with ≥80% non-missing values. If fewer than *n*_Features_ viruses in *D*_*j*_ satisfy this criterion, do not grow decision trees for this dataset
  ○ Bootstrap sample (with replacement) both the viruses and antibody responses
  ○ Data are analyzed in log_10_ and row-centered on the features (i.e., for each antibody response in either the training set *D*_*j*_ or testing set *D*_0_, subtract the mean of the log_10_[titers] for the *n*_Features_ viruses using all non-missing measurements) to account for systematic shifts between datasets. Row-centering is undone once decision trees make their predictions by adding the serum-dependent mean
  ○ Compute the cross-validation root-mean-square error (RMSE, *σ*_Training_) of each tree using the remaining 1 − *f*_Samples_ fraction of samples in *D*_*j*_
2. Predict the (un-row-centered) values of *V*_0_ in *D*_0_ using the *n*_BestTrees_ = 5 decisions trees with the lowest *σ*_Training_
  ○ Trees only make predictions in *D*_0_ where *all n*_Features_ are non-missing
  ○ Predict *μ*_*j*_ ± *σ*_*j*_ for each antibody response
    ▪ *μ*_*j*_ = (mean value for *n*_BestTrees_ predictions)
    ▪ *σ*_*j*_ = $f_{D_{j}\to D_{0}}$(mean *σ*_Training_ for *n*_BestTrees_ trees), where the transferability $f_{D_{j}\to D_{0}}$ is computed by predicting *V*_*j*,1_, *V*_*j*,2_… in *D*_0_ using *D*_*j*_ (see Box 2)
3. Combine predictions for *V*_0_ in *D*_0_ with all other datasets {*D*_*j*_} using $\frac{\Sigma_{j}\;\left(\mu_{j}/\sigma_{j}^{2}\right)}{\Sigma_{j}\;\left(1/\sigma_{j}^{2}\right)}\pm \frac{1}{{\left[\Sigma_{j}\left(1/\sigma_{j}^{2}\right)\right]}^{1/2}}$

One potential pitfall of this approach is that the estimated error *σ*_Training_ derived from dataset 2 will almost always underestimate the true error for these predictions (*σ*_Actual_) in dataset 1 because the antibody responses in both studies may be very distinct (e.g., sera collected decades apart or from people/animals with different infection histories).

To correct for this effect, we estimate an upper bound for *σ*_Actual_ by computing the *transferability f*_2→1_(*x*), which quantifies the accuracy of a relation found in dataset 2 (e.g., *V*_0_ = *V*_1_ + *V*_2_, although complex non-linear relations are allowed) when applied to dataset 1. More precisely, if a relation has error *σ*_Training_ in dataset 2 and *σ*_Actual_ in dataset 1, then the transferability gives an upper bound, *f*_2→1_(*σ*_Training_ from dataset 2) ≥ *σ*_Actual_ in dataset 1, that holds for the majority of decision trees. Thus, a low *f*_2→1_(*σ*_Training_ from dataset 2) guarantees accurate predictions.

To calculate the transferability *f*_2→1_, we repeat the above algorithm, but rather than inferring values for *V*_0_, we predict each of the overlapping viruses *V*_1_-*V*_*n*_ measured in *both* datasets whose *σ*_Training_ and *σ*_Actual_ can be directly computed (Figure 2D; Box 2). We found that transferability was well characterized by a simple linear relationship (Figure S1; note that *f*_2→1_ represents an upper bound and not an equality). Finally, we apply this relation to the training error for virus *V*_0_ to estimate prediction error in dataset 1, *σ*_Predict_ ≡ *f*_2→1_(*σ*_Training_). In this way, both values and errors for *V*_0_ are inferred using a generic, data-driven approach that can be applied to diverse datasets.

Box 2 **Computing the transferability fDj→D0 between datasets**

*Input*:

• Datasets {*D*_*j*_} that collectively include viruses *V*_1_, *V*_2_… Each virus must be included in at least two datasets

*Steps*:

• For each dataset *D*_0_ in {*D*_*j*_}, for each virus *V*_0_ in *D*_0_, for every other dataset *D*_*j*_ containing *V*_0_
• Create *n*_Tree__s_ = 50 decision trees predicting *V*_0_ based on *n*_Features_ = 5 other viruses, as described in Box 1
• For each tree, store the following:
  ○ *D*_0_, *V*_0_, and *D*_*j*_ used to construct the tree
  ○ Viruses used to train the tree
  ○ RMSE *σ*_Training_ on the 1-*f*_Samples_ samples in *D*_*j*_
  ○ Predictions of *V*_0_’s values in *D*_0_
  ○ True RMSE *σ*_Actual_ of these predictions for *V*_0_ in *D*_0_
• When predicting *V*_0_ using *D*_*j*_*→D*_0_ in Box 1, we compute $f_{D_{j}\to D_{0}}$ between *σ*_Training_ and *σ*_Actual_ by predicting the other viruses *V*_1_, *V*_2_…*V*_*n*_ that overlap between *D*_*j*_ and *D*_0_ (making sure to only use decision trees that exclude the withheld *V*_0_)
  ○ From the forest of decision trees above, find the top 10 trees for each virus predicted between *D*_*j*_*→D*_0_ and plot *σ*_Training_ vs. *σ*_Actual_ for all trees (see Figure S1)
  ○ Find the best-fit line using perpendicular offsets, *y* = *ax*+*b* where *x* = *σ*_Training_ and *y* = *σ*_Actual_. Since there is scatter about this best-fit line, and because it is better to overestimate rather than underestimate error, we add a correction factor *c*=(RMSE between *σ*_Actual_ and *ax*+*b*). Lastly, we expect that a decision tree’s error in another dataset will always be at least as large as its error on the training set (*σ*_Actual_≥*σ*_Training_), and hence we define $f_{D_{j}\to D_{0}}$ = max(*aσ*_Training_+*b*+*c*, *σ*_Training_). This max term is important in a few cases where $f_{D_{j}\to D_{0}}$ has a very steep slope but some decision trees have small *σ*_Training_
  ○ Datasets with high transferability will have $f_{D_{j}\to D_{0}}$(*σ*_Training_)≈*σ*_Training_, meaning that viruses can be removed from *D*_0_ and accurately inferred from *D*_*j*_*.* In contrast, two datasets with low transferability will have a nearly vertical line, *∂*$f_{D_{j}\to D_{0}}$/*∂σ*_Training_≫1, signifying that viruses will be poorly predicted between these studies
  ○ In the chord diagrams (Figures 4B and 5B), the width of the arc between Dataset *D*_*j*_ and *D*_0_ is proportional to (*∂*$f_{D_{j}\to D_{0}}$/*∂σ*_Training_)^−1^

### Leave one out: Inferring virus behavior without a single measurement

To assess matrix completion across studies, we applied it to three increasingly difficult scenarios: (1) between two highly similar human vaccination studies, (2) between a human infection and human vaccination study, and (3) between a ferret infection and human vaccination study. We expected prediction accuracy to decrease as the datasets become more distinct, resulting in both a larger error (*σ*_Actual_) and larger estimated uncertainty (*σ*_Predict_).

For these predictions, we utilized the Fonville influenza datasets consisting of six studies: four human vaccination studies (dataset_Vac,1–4_), one human infection study (dataset_Infect,1_), and one ferret infection study (dataset_Ferret_).^20^ In each study, sera were measured against a panel of H3N2 viruses using HAI. Collectively, these studies contained 81 viruses, and each virus was measured in at least two studies.

We first predicted values for the virus *V*_0_ = A/Auckland/5/1996 in the most recent vaccination study (dataset_Vac,4_) using data from another vaccination study (dataset_Vac,3_) carried out in the preceding year and in the same geographic location (Table S1). After training our decision trees, we found that the two studies had the best possible transferability (*σ*_Predict_ = *f*_Vac,3→Vac,4_(*σ*_Training_) ≈ *σ*_Training_), suggesting that there is no penalty in extrapolating virus behavior between these datasets. More precisely, if there exist five viruses, *V*_1_–*V*_5_, that can accurately predict *V*_0_’s measurements in dataset_Vac,3_, then *V*_1_–*V*_5_ will predict *V*_0_ equally well in dataset_Vac,4_.

**Figure 3:**
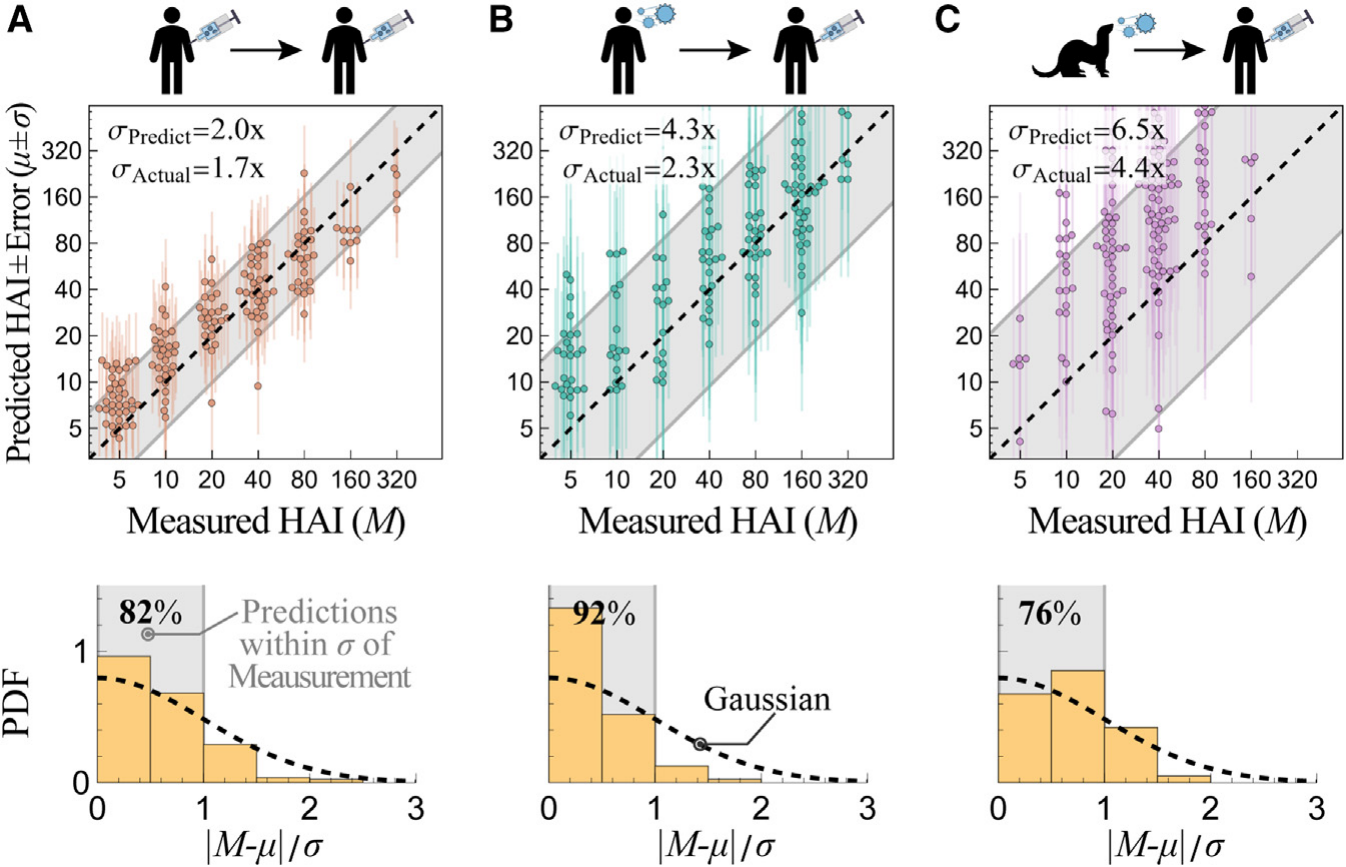
Predicting virus behavior between two datasets Example predictions between two Fonville studies. Top: plots comparing predicted and withheld HAI measurements (which take the discrete values 5, 10, 20…). Estimated error is shown in two ways: (1) as vertical lines emanating from each point and (2) by the diagonal gray bands showing *σ*_Predict_. Bottom: histograms of the standardized absolute prediction errors compared with a standard folded Gaussian distribution (black dashed line). The fraction of predictions within 1.0*σ* are shown at the top left, which can be compared with the expected 68% for the standard folded Gaussian distribution. (A) Predicting A/Auckland/5/1996 between two human vaccination studies (datasets_Vac,3→Vac,4_). (B) Predicting A/Netherlands/620/1989 between a human infection and human vaccination study (datasets_Infect,1→Vac,4_). (C) Predicting A/Victoria/110/2004 between a ferret infection and human vaccination study (datasets_Ferret→Vac,4_).

Indeed, we found multiple such decision trees that predicted *V*_0_’s HAI titers with *σ*_Predict_ = 2.0-fold uncertainty, meaning that each titer *t* is expected to lie between *t*/2 and *t*·2 with 68% probability (or, equivalently, that log_10_(*t*) has a standard deviation of log_10_(2)) (top panel in Figure 3A, gray bands represent *σ*_Predict_). Notably, this estimated uncertainty closely matched the true error *σ*_Actual_ = 1.7-fold. To put these results into perspective, the HAI assay has roughly 2-fold error (i.e., repeated measurements differ by 2-fold 50% of the time and by 4-fold 10% of the time; STAR Methods), implying that these predictions are as good as possible given experimental error.

When we inferred every other virus between these vaccine studies (datasets_Vac,3→Vac,4__)_, we consistently found the same highly accurate predictions: *σ*_Predict_≈*σ*_Actual_ ≈ 2-fold (Figure S2A). As an alternative way of quantifying error, we plotted the distribution of predictions within 0.5, 1.0, 1.5… standard deviations from the measurement, which we compare against a folded Gaussian distribution (Figure 3A, bottom). For example, 82% of predictions were within 1 standard deviation, somewhat larger than the 68% expected for a Gaussian, confirming that prediction error was slightly overestimated.

We next predicted values for *V*_0_ = A/Netherlands/620/1989 between a human infection and vaccination study (dataset_Infect,1→Vac,4_). In this case, the predicted values were also highly accurate with true error *σ*_Actual_ = 2.3-fold (Figure 3B; remaining viruses predicted in Figure S2B). When quantifying the uncertainty of these predictions, we found worse transferability of virus behavior (*f*_Infect,1→Vac,4_(*σ*_Training_) ≈ 2.8*σ*_Training_, where the larger prefactor of 2.8 indicates less transferability; STAR Methods), and hence we overestimated the prediction error as *σ*_Predict_ = 4.3-fold. Last, when we predicted values for *V*_0_ = A/Victoria/110/2004 between a ferret infection and human vaccination study (dataset_Ferret→Vac,4_), our predictions had a larger true error, *σ*_Actual_ = 4.4-fold (Figure 3C), than the inferences between human data, as expected. Moreover, poor transferability between these datasets led to a poorer guarantee of prediction accuracy, *σ*_Predict_ = 6.5-fold, indicative of larger variability when predicting between ferret and human data.

Importantly, we purposefully constructed *σ*_Predict_ to overestimate *σ*_Actual_ when datasets *X* and *Y* exhibit disparate behaviors, since matching the average distribution of *σ*_Predict_ to *σ*_Actual_ could lead to an unwanted underestimation of the true error. With our approach, a low *σ*_Predict_ guarantees accurate predictions. As we show in the following section, the estimated values and error become more precise when we use multiple datasets to infer virus behavior.

### Combining influenza datasets to predict 200,000 measurements with ≤3-fold error

When multiple datasets are available to predict virus behavior in dataset 1, we obtain *predictions ± errors* (*μ*_*j*_ ± *σ*_*j*_) from dataset 2→1, dataset 3→1, dataset 4→1… These predictions and their errors are combined using the standard Bayesian approach as

(Equation 1) $$\frac{\Sigma_{j}\;\left(\mu_{j}/\sigma_{j}^{2}\right)}{\Sigma_{j}\;\left(1/\sigma_{j}^{2}\right)}\pm \frac{1}{{\left[\Sigma_{j}\left(1/\sigma_{j}^{2}\right)\right]}^{1/2}.}$$

The uncertainty term in this combined prediction has two key features. First, adding any additional dataset (with predictions *μ*_*k*_ ± *σ*_*k*_) can only *decrease* the uncertainty. Second, if a highly uninformative dataset is added (with *σ*_*k*_→∞), it will negligibly affect the cumulative prediction. Therefore, as long as the uncertainty estimates are reasonably precise, datasets do not need to be prescreened before matrix completion, and adding more datasets will always result in lower uncertainty.

**Figure 4:**
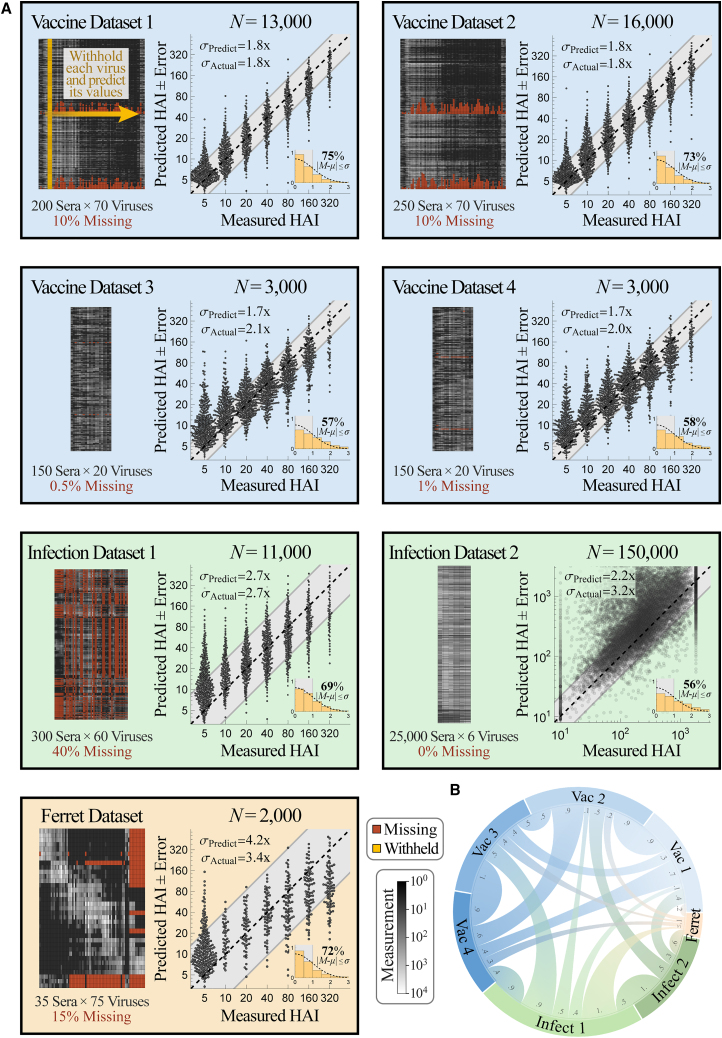
Validating prediction ± error quantification across 200,000 measurements (A) We combined seven influenza datasets spanning human vaccination studies (blue boxes), human infection studies (green), and a ferret infection study (orange). Each virus in every dataset was withheld and predicted using the remaining data (shown schematically in gold in the top left box). We display each dataset (left; missing values in dark red and measurements in grayscale) and the collective predictions for all viruses in that dataset (right; gray diagonal bands show the average predicted error *σ*_Predict_). The total number of predictions *N* from each dataset is shown above the scatterplots; when this number of points is too great to show, we subsampled each distribution evenly while maintaining its shape. The inset at the bottom right of each plot shows the probability density function (PDF) histogram of error measurements (y axis) that were within 0.5*σ*, 1.0*σ*, 1.5*σ*… (*x*-axis) compared with a standard folded Gaussian distribution (black curve). The fraction of predictions within 1.0*σ* is explicitly written and can be compared with the expected 68% for a standard folded Gaussian. (B) Chord diagram representing the transferability between datasets. For each arc connecting dataset *X*→*Y*, transferability is shown near the outer circle of *Y*, with larger width representing greater transferability (Figures S1 and S4; STAR Methods).

To test the accuracy of combining multiple datasets, we performed leave-one-out analysis using all six Fonville studies, systematically withholding every virus in each dataset (311 virus-dataset pairs) and predicting the withheld values using all remaining data. Each dataset measured 35–300 sera against 20–75 viruses (with 81 unique viruses across all 6 studies) and had 0.5%–40% missing values (Figure 4A).

Collectively, we predicted the 50,000 measurements across all datasets with a low error of *σ*_Actual_ = 2.1-fold (between the measured value and the left-hand side of Equation 1). Upon stratifying these predictions by dataset, we found that the four human vaccination studies were predicted with the highest accuracy (datasets_Vac,1–4_, *σ*_Actual_ ≈ 2-fold), while the human infection study had slightly worse accuracy (dataset_Infect,1_, *σ*_Actual_ = 2.7-fold) (Figure 4A). Remarkably, even the least accurate human → ferret predictions had ≤4-fold error on average (*σ*_Actual_ = 3.4-fold), demonstrating the potential for these cross-study inferences. As negative controls, permutation testing as well as predictions based solely on virus sequence similarity led to nearly flat predictions with substantially larger error (Figure S3).

In addition to accurately predicting these values, the estimated error closely matched the true error in every human study (*σ*_Predict_ ≈ *σ*_Actual_, datasets_Vac,1–4_ and dataset_Infect,1_). The uncertainty of the ferret predictions was slightly overestimated (*σ*_Predict_ = 4.2-fold, dataset_Ferret_); mathematically, this occurs because the upper envelope of *σ*_Training_-vs-*σ*_Actual_ is steep, making *σ*_Actual_ difficult to precisely determine (Figure S1).

We visualize the transferability between datasets using a chord diagram (Figure 4B), where wider bands connecting datasets_*X*↔*Y*_ represent larger transferability (Figure S4; STAR Methods). As expected, there was high transferability between the human vaccine studies carried out in consecutive years (datasets_Vac,1↔Vac,2_ and datasets_Vac,3↔Vac,4_, Table S1) but generally less transferability across vaccine studies more than 10 years apart (datasets_Vac,1↔Vac,3_, datasets_Vac,1↔Vac,4_, datasets_Vac,2↔Vac,3_, or datasets_Vac,2↔Vac,4_).

Transferability is not necessarily symmetric because virus inhibition in dataset *X* could exhibit all patterns in dataset *Y* (leading to high transferability from *X*→*Y*) along with unique patterns not seen in dataset *Y* (resulting in low transferability from *Y*→*X*). For example, all human datasets displayed small transferability to the ferret data, whereas the ferret dataset accurately predicts the human dataset_Infect,1_; this suggests that the ferret responses show some patterns present in the human data but also display unique phenotypes. As another example, the human infection study carried out from 2007–2012 had high transferability from the human vaccine studies conducted in 2009 and 2010 (dataset_Vac,3/4→Infect,1_) but showed smaller transferability in the reverse direction.

To show the generality of this approach beyond H3N2 HAI data, we predicted H1N1 virus neutralization across two monoclonal antibody datasets, finding an error *σ*_Actual_ = 3.0–3.6-fold across measurements spanning two orders of magnitude (Figure S5). While these serum and monoclonal antibody results lay the foundation to compare datasets and quantify the impact of a person’s age, geographic location, and other features on the antibody response, they are not exhaustive characterizations; for example, additional human datasets may be able to more accurately predict these ferret responses. The strength of this approach lies in the fact that cross-study relationships are learned in a data-driven manner. As more datasets are added, the number of predictions between datasets increases, while the uncertainty of these predictions decreases.

### Versatility of matrix completion: Predicting values from a distinct assay using only 5 overlapping viruses

To test the limits of our approach, we used the Fonville datasets to predict values from a large-scale serological dataset by Vinh et al.,^25^ where *only 6* influenza viruses were measured against 25,000 sera. This exceptionally long and skinny matrix is challenging for several reasons. First, after entirely withholding a virus, only 5 other viruses remain to infer its behavior. Furthermore, only 4 of the 6 Vinh viruses had exact matches in the Fonville dataset; given this small virus panel, we utilized the remaining 2 viruses by associating them with the closest Fonville virus based on their hemagglutinin sequences (STAR Methods; sequences available in GitHub repository). Associating functionally distinct viruses will result in poor transferability, and hence the validity of matching nearly homologous viruses can be directly assessed by comparing the transferability with or without these associations.

Second, the Vinh study used protein microarrays to measure serum binding to the HA1 subunit that forms the hemagglutinin head domain. While HAI also measures how antibodies bind to this head domain, such differences in the experimental assay could lead to fundamentally different patterns of virus inhibition, resulting in smaller transferability and higher error.

Third, there were only 1,200 sera across all Fonville datasets, and hence predicting the behavior of 25,000 Vinh sera will be impossible if they all exhibit distinct phenotypes. Indeed, any such predictions would only be possible if this swarm of sera are highly degenerate, the behavior of each Vinh virus can be determined from the remaining 5 viruses, and these same relations can be learned from the Fonville data. Last, we note one superficial difference: the Vinh data span a continuum of values, while the Fonville data take on discrete 2-fold increments, although this feature does not affect our algorithm.

After growing a forest of decision trees to establish the transferability between the Fonville and Vinh datasets (Figure S1), we predicted the 25,000 serum measurements for all 6 Vinh viruses with an average *σ*_Actual_ = 3.2-fold error, demonstrating that even a small panel containing 5 viruses can be expanded to predict the behavior of additional strains (Figure 4A, dataset_Infect,2_).

Notably, 5 of these 6 viruses (which all circulated between 2003 and 2011) had a very low *σ*_Predict_≈*σ*_Actual_ ≈ 2- to 3-fold error (Figure S6). The final Vinh virus circulated three decades earlier (in 1968), and its larger prediction error was underestimated (*σ*_Actual_ = 9.3-fold, *σ*_Predict_ = 3.8-fold). This highlights a shortcoming of any matrix completion algorithm; namely, that when a dataset contains one exceptionally distinct column (i.e., one virus circulating 30 years before all other viruses), its values will not be accurately predicted. These predictions would have improved had these six viruses been sampled uniformly between 1968 and 2011.

### Leave multi out: Designing a minimal virus panel that maximizes the information gained per experiment

Given the accuracy of leave-one-out analysis and that only 5 viruses are needed to expand a dataset, we reasoned that these studies contain a plethora of measurements that could have been inferred by cross-study predictions. Pushing this to the extreme, we combined the Fonville and Vinh datasets and performed *leave-multi-out analysis*, where multiple viruses were simultaneously withheld and recovered. Future studies seeking to measure any set of viruses, *V*_1_–*V*_*n*_, can use a similar approach to select the minimal virus panel that predicts their full data.

**Figure 5:**
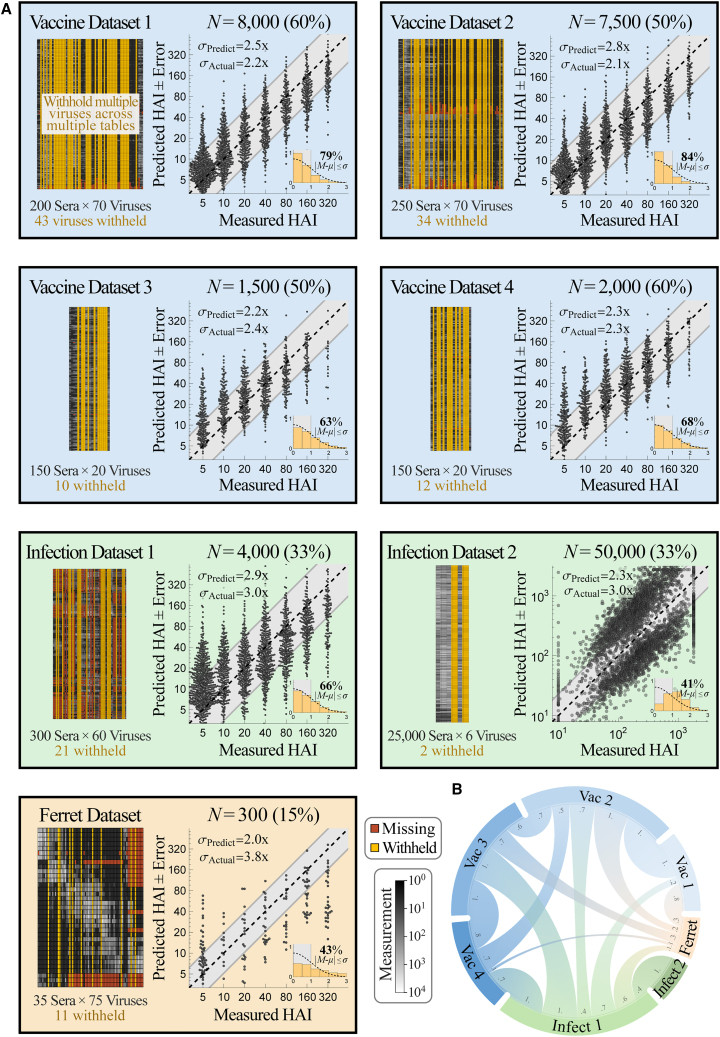
Simultaneously predicting 133 viruses withheld from multiple datasets (A) Viruses were concurrently withheld from each dataset (left, gold columns), and their 70,000 values were predicted using the remaining data. We withheld as many viruses as possible while still estimating a low error of *σ*_Predict_ ≤ 4-fold (blinding ourselves to actual measurements), and indeed, the actual prediction error was smaller than 4-fold in every dataset. As in Figure 4, plots and histograms show the collective predictions and error distributions. The plot label enumerates the number of concurrent predictions (and percent of data predicted). (B) Chord diagram representing the transferability between datasets after withholding the viruses. For each arc connecting datasets *X*→*Y*, transferability is shown near the outer circle of *Y*, with larger width representing greater transferability (Figures S1 and S4; STAR Methods).

In the present search, we sought the minimum viruses needed to recover all Fonville and Vinh measurements with ≤4-fold error; we chose this threshold because it lets us remove dozens of viruses while being much smaller than the 1,000-fold range of the data. A virus was randomly selected from a dataset and added to the withheld list when its values, and those of all other withheld viruses, could be predicted with *σ*_Predict_ ≤ 4-fold (without using *σ*_Actual_ to confirm these predictions; STAR Methods). In this way, 133 viruses were concurrently withheld, representing 15%–60% of the virus panels from every dataset or a total of *N* = 70,000 measurements (Figure 5A).

Even with this hefty withheld set, prediction error was only slightly larger than during leave-one-out analysis (*σ*_Actual_ between 2.1- to 3.0-fold for the human datasets and *σ*_Actual_ = 3.8-fold for the ferret data). This small increase is due to two competing factors. On one hand, prediction is far harder with fewer viruses. At the same time, our approach specifically withheld the most “redundant” viruses that could be accurately estimated (with *σ*_Predict_ ≤ 4-fold). These factors mostly offset one another so that the 70,000 measurements exhibited the desired *σ*_Actual_ ≤ 4-fold.

The transferability between datasets, computed without the withheld viruses, was similar to the transferability between the full datasets (Figure 5B). Some connections were lost when there were <5 overlapping viruses between datasets, while other connections were strengthened when the patterns in the remaining data became more similar across studies. Notably, the ferret data now showed some transferability from vaccination datasets_Vac,1/2_, which resulted in smaller estimated error (*σ*_Predict_ = 2.9-fold) than in our leave-one-out analysis. This emphasizes that transferability depends on the specific viruses and sera examined and that some parts of the Fonville human dataset can better characterize ferret data. While this uncertainty underestimated the true error of the ferret predictions (*σ*_Actual_ = 3.8-fold), both types of errors were within the desired 4-fold error threshold. Moreover, in all six human datasets, the estimated uncertainty *σ*_Predict_ closely matched the true error *σ*_Actual_, demonstrating significant potential in predicting virus behavior, especially between datasets of the same type such as human vaccine or infection studies.

### Expanding datasets with 2 × 10^6^ new measurements reveals a tradeoff between serum potency and breadth

**Figure 6:**
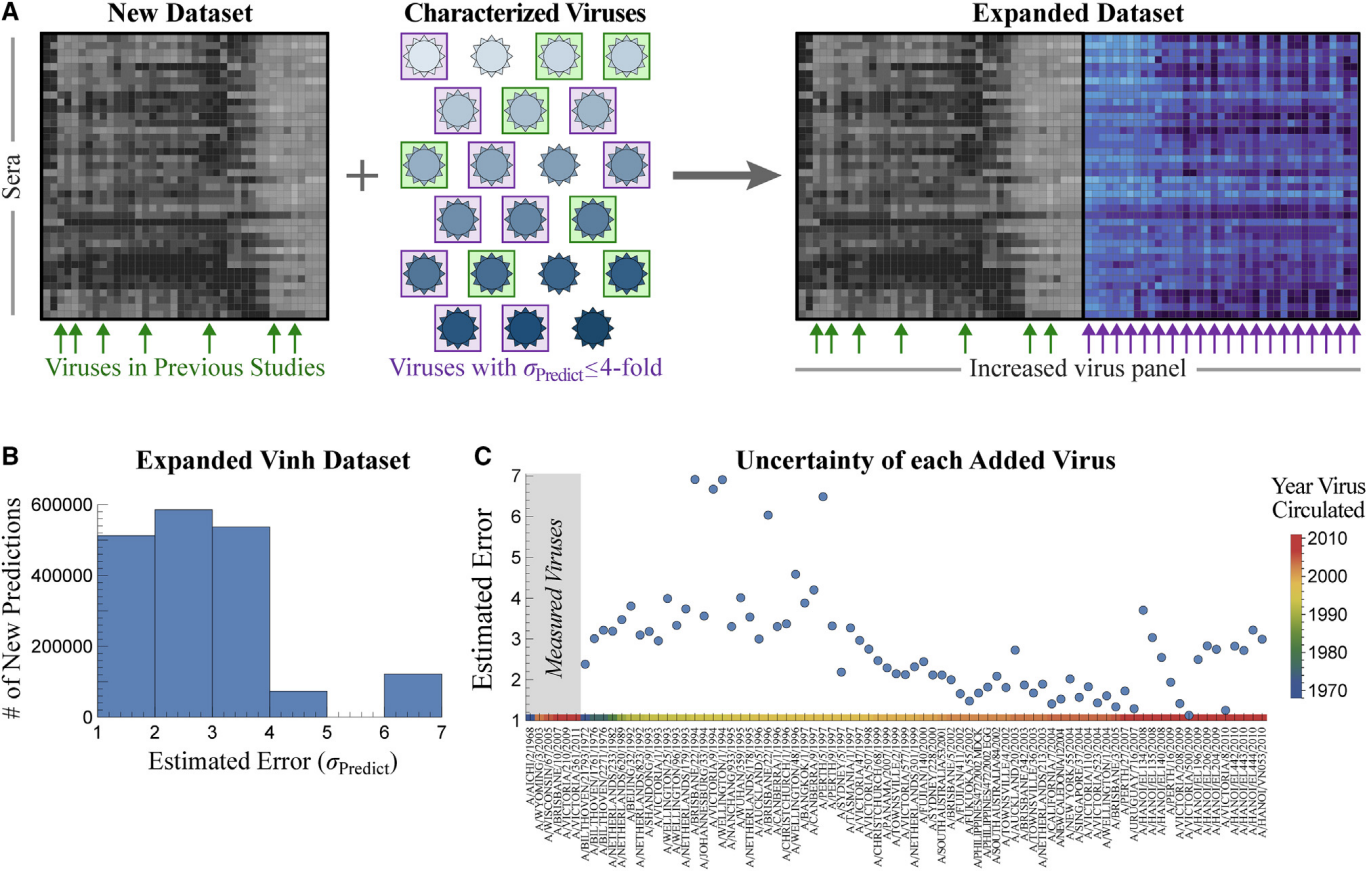
Expanding the Vinh dataset with 75 additional viruses (A) If a new study contains at least 5 previously characterized viruses (green boxes and arrows), we can predict the behavior of all previously characterized viruses in the new dataset. Those with an acceptable error (e.g., ≤4-fold error boxed in purple) are used to expand the dataset. (B) Distribution of the estimated uncertainty *σ*_Predict_ when predicting how each Fonville virus inhibits the 25,000 Vinh sera. Most viruses are estimated with ≤4-fold error. (C) Estimated uncertainty of each virus. The six viruses on the left represent the Vinh virus panel. Colors at the bottom represent the year each virus circulated.

In the previous section, we combined datasets to predict serum-virus HAI titers, validating our approach on 200,000 existing measurements. Future studies can immediately leverage the Fonville datasets to expedite their efforts. If a new dataset contains at least 5 Fonville viruses (green arrows/boxes in Figure 6A), then HAI values ± errors for the remaining Fonville viruses can be predicted. Viruses with an acceptably low error (purple in Figure 6A) can be added without requiring any additional experiments.

To demonstrate this process, we first focus on the Vinh dataset, where expansion will have the largest impact because the Vinh virus panel is small (6 viruses), but its serum panel is enormous (25,000 sera). By predicting the interactions between these sera and all 81 unique Fonville viruses, we add 2,000,000 new predictions (more than 10× the number of measurements in the original dataset).

For each Fonville virus *V*_0_ that was not measured in the Vinh dataset, we grew a forest of decision trees as described above, with the minor modification that the 5 features were restricted to the Vinh viruses to enable expansion. The top trees were combined with the transferability functions (Figure S1) to predict the values ± errors for *V*_0_ (Figure S7).

The majority of the added Fonville viruses (67 of 75) had tight predictions of *σ*_Predict_ ≤ 4-fold (Figure 6B). As expected, viruses circulating around the same time as the Vinh panel (1968 or 2003–2011) tended to have the lowest uncertainty, whereas the furthest viruses from the 1990s had the largest uncertainty (Figure 6C). To confirm these estimates, we restricted the Fonville datasets to these same 6 viruses and expanded out, finding that any virus with *σ*_Predict_ ≤ 6-fold prediction error (which applies to nearly all Vinh predictions) had a true error *σ*_Actual_ ≤ 6-fold (Figure S8). We similarly expanded the Fonville datasets, adding 175 new virus columns across the six studies (Figure S7; extended datasets provided on GitHub). In addition, dimensionality reduction via uniform manifold approximation and projection (UMAP) recovered a linear trend from the oldest to newest viruses in both the Fonville and Vinh datasets; this trend is especially noteworthy in the latter case because we did not supply the circulation year for the 75 inferred viruses, yet we can discern its impact on the resulting data (Figure S9).

**Figure 7:**
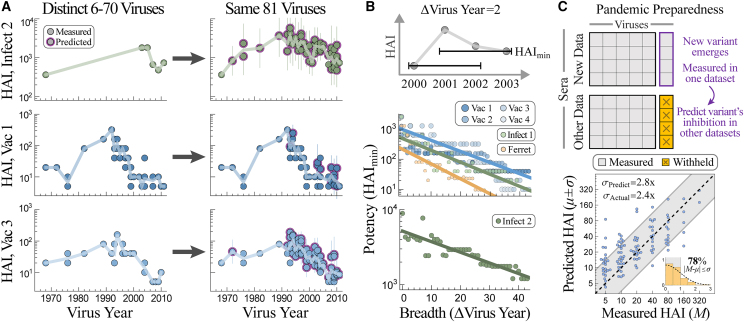
Applications of cross-study predictions (A) We predict HAI titers for 25,000 sera against the same set of 81 viruses, providing high-resolution landscapes that can be directly compared against each other. Representative responses are shown for dataset_Infect,2_ (top, serum 5130165 in GitHub), dataset_Vac,1_ (center, subject 525), and dataset_Vac,3_ (bottom, subject A028). (B) Tradeoff between serum breadth and potency, showing that viruses spaced apart in time are harder to simultaneously inhibit. For every study and each possible set of viruses circulating within Δvirus years of each other, we calculate the highest HAI_min_ (i.e., a serum exists with HAI titers ≥ HAI_min_ against the entire set of viruses). (C) Top: when a new variant emerges and is measured in a single study, we can predict its titers in all previous studies with ≥5 overlapping viruses. Bottom: example predicting how the newest variant in the newest vaccine dataset is inhibited by sera from a previous vaccine study (datasets_Vac,4→Vac,3_).

For each Vinh serum, this expansion fills in the 3.5-decade gap between 1968 and 2003 by predicting 47 additional viruses, as well as adding another 28 measurements between 2003 and 2011 (Figure 7A, new interactions highlighted in purple). We also predicted dozens of new viruses in the vaccine studies, and for some sera this increased resolution revealed a more jagged landscape than what was apparent from the direct measurements (Figure 7A). Although HAI titers tend to be similar for viruses circulating around the same time, exceptions do arise (e.g., A/Tasmania/1/1997 vs. A/Perth/5/1997 as well as A/Hanoi/EL201/2009 vs. A/Hanoi/EL134/2008 had >4-fold difference in their predicted titers), and our expanded data reveal these functional differences between variants.

The expanded data also enable a direct comparison of sera across studies, something that is exceedingly difficult with the original measurements given that none of the 81 viruses were in all 7 datasets. Figure 7A shows that an antibody response may be potent against older strains circulating before 2000 but weak against newer variants (bottom), highly specific against strains from 1980–2000 with specific vulnerabilities to viruses from 1976 (center), or relatively uniformly against the entire virus panel (top).

We next used the expanded data to probe a fundamental but often unappreciated property of the antibody response; namely, the tradeoff between serum potency and breadth. Given a set of viruses circulating within Δvirus years of each other (the top of Figure 7B shows an example with Δvirus years = 2), how potently can a serum inhibit all of these variants simultaneously? For any set of viruses spanning Δvirus years, we computed HAI_min_ (the minimum titer against this set of viruses) for each serum and plotted the maximum HAI_min_ in each dataset (Figure 7B). (While children born after the earliest circulating strains may have artificially smaller HAI_min_, every dataset contains adults born before the earliest strain, and we only report the largest potency in each study.) We find that HAI_min_ decreases with Δvirus years, demonstrating that it is harder to simultaneously inhibit more diverse viruses. This same tradeoff was seen for monoclonal antibodies,^29^^,^^30^ and it suggests that efforts geared toward finding extremely broad and potentially universal influenza responses may run into an HAI ceiling.

### Toward pandemic preparedness

When two studies have high transferability, each serves as a conduit to rapidly propagate information. For example, if a new variant *V*_0_ emerges this year, the most pressing question is whether our preexisting immunity will inhibit this new variant or whether it is sufficiently distinct to bypass our antibody response.

Traditionally, antigenic similarity is measured by infecting ferrets with prior circulating strains and measuring their cross-reactivity to the new variant, yet the above analysis (and work by many others^31^^,^^32^) shows that ferret↔human inferences can be poor. Instead, we can rapidly assess the inhibition of *V*_0_ in multiple existing human cohorts that measured HAI against viruses *V*_1_–*V*_5_ by measuring a single additional human cohort against *V*_0_–*V*_5_ and then predicting *V*_0_’s titers in all other studies. As an example, consider the more recent virus strain in the latest vaccine dataset (A/Perth/16/2009 from vaccine study 4, carried out in 2010, around the time this variant emerged). Our framework predicts how all individuals in vaccine study 3 inhibit this variant with *σ*_Actual_ = 2.4-fold error (Figure 7C).

Another recent application of pandemic preparedness tested the breadth of an influenza vaccine containing H1N1 A/Michigan/45/2015 by measuring the serum response against one antigenically distinct H1N1 A/Puerto Rico/8/1934 strain.^33^ Inferring additional virus behavior would provide greater resolution into the coverage and potential holes of an antibody response. As shown in Figure 7A, ≈5 measurements can extrapolate serum HAI against viruses circulating in multiple decades, providing this needed resolution from a small number of interactions.

### Matrix completion via nuclear norm minimization poorly predicts behavior across studies

In this final section, we briefly contrast our algorithm against singular value decomposition (SVD)-based approaches, such as nuclear norm minimization (NNM), which are arguably the simplest and best-studied matrix completion methods. With NNM, missing values are filled by minimizing the sum of singular values of the completed dataset.

To compare our results, we reran our leave-multi-out analysis from Figure 5, simultaneously withholding 133 viruses and predicting their values using an established NNM algorithm from Einav and Cleary.^7^ The resulting predictions were notably worse, with *σ*_Actual_ between 3.4 and 5.4-fold.

Because of two often neglected features of NNM, we find that our approach significantly outperforms this traditional route of matrix completion in predicting values for a completely withheld virus column. First, NNM is asymmetrical when predicting large and small values for a withheld virus. Consider a simple noise-free example where one virus’s measurements are proportional to another’s, (virus 2’s values) = *m* × (virus 1’s values) (Figure S10A shows *m* = 5). Surprisingly, even if provided with one perfect template for these measurements, NNM incorrectly predicts that (virus 2’s values) = (virus 1’s values) for any *m* ≥ 1 (Figure S10B). This behavior is exacerbated when multiple datasets are combined, emphasizing that NNM can catastrophically fail for very simple examples (Figures S10C and S10D). This artifact can be alleviated by first row-centering a dataset (subtracting the mean of the log_10_[titers] for each serum in Figure 2A), as in Box 1.

Even with row-centering, a second artifact of NNM is that large swaths of missing values can skew matrix completion because relationships are incorrectly inferred between these missing values. Intuitively, all iterative NNM algorithms must initialize the missing entries (often either with 0 or the row/column means), so that after initialization, two viruses with very different behaviors may end up appearing identical across their missing values. For example, suppose we want to predict values for virus *V*_0_ from dataset *X*→*Y* and that “useful” viruses *V*_1_–*V*_4_ behave similarly to *V*_0_ in datasets *X* and *Y*. On the other hand, “useless” viruses *V*_5_–*V*_8_ are either not measured in dataset 2 or are measured against complementary sera; moreover, these viruses show very different behavior from *V*_0_ in dataset 1 (Figures S10E and S10F show a concrete example from Fonville). Ideally, matrix completion should ignore *V*_5_–*V*_8_ (given that they do not match *V*_0_ in dataset 2) and only use *V*_1_–*V*_4_ to infer *V*_0_’s values in dataset 1. In practice, NNM using *V*_0_–*V*_8_ results in poor predictions (Figures S10E and S10F). This behavior is disastrous for large serological datasets, where there can be >50% missing values when datasets are combined.

Our algorithm was constructed to specifically avoid both artifacts. First, we infer each virus’s behavior using a decision tree on row-centered data that does not exhibit the asymmetry discussed above. Second, we restrict our analysis to features that have ≥80% observed measurements to ensure that patterns detected are based on measurements rather than on missing data.

As another point of comparison, consider the leave-one-out predictions of the six Vinh viruses using the Fonville datasets. Whereas our algorithm yields tight predictions across the full range of values (Figure S6), NNM led to a nearly flat response, with all 25,000 sera incorrectly predicted to be the mean of the measurements (see Figure S11 in Einav and Cleary^7^). In addition, we utilized an existing SVD-based matrix completion method that quantifies the prediction uncertainty for each entry under the assumption that values are randomly missing from a dataset.^18^ Applying this method to the Fonville datasets resulted in predictions whose actual error was >20-fold larger than the estimated error, emphasizing the need for frameworks that specifically handle structured missing data.^34^

## Discussion

By harnessing the wealth of previously measured antibody-virus interactions, we can catapult future efforts and design experiments that are far larger in size and scope. Here, we developed an algorithm that leverages patterns in HAI data to predict how a virus measured in one study would inhibit sera from another study without requiring any additional experiments. Even when the original studies only had a few overlapping viruses, the expanded datasets can be directly compared using all variants.

While it is understood that sera cross-react, exhibiting similar inhibition against nearly homologous variants, it is unclear whether there are universal relationships that hold across datasets. We introduce the notion of transferability to quantify how accurately local relations within one dataset map onto another dataset (Figure 4B; STAR Methods).^35^ Transferability is based on the functional responses of viruses, and it does not require side information, such as virus sequence or structure, although future efforts should quantify how incorporating such information reduces prediction error. In particular, incorporating sequence information could strengthen predictions when virus panels have little direct overlap but contain many nearly homologous variants.

It is rarely clear *a priori* when two datasets can inform one another; will differences in age, geographic location, or infection history between individuals fundamentally change how they inhibit viruses?^36^^,^^37^^,^^38^ Transferability directly addresses these questions. Through this lens, we compared the Fonville and Vinh studies, which utilized different assays, had different dynamic ranges, and used markedly different virus panels.^20^^,^^25^ We found surprisingly large transferability between human infection and vaccination studies. For example, vaccine studies from 1997/1998 (dataset_Vac,1/2_) were moderately informed by the Vinh infection study from 2009–2015 (dataset_Infect,2_), even though none of the Vinh participants had ever been vaccinated (Figure 4B). Conversely, both infection studies we analyzed were well informed by at least one vaccine study (e.g., dataset_Infect,1_ was most informed by datasets_Vac,3/4_).

These results demonstrate that diverse cohorts can inform one another. Hence, instead of thinking about each serum sample as being entirely unique, large collections of sera may often exhibit surprisingly similar inhibition profiles. For example, the 1,200 sera in the Fonville datasets predicted the behavior of the 25,000 Vinh sera with ≤2.5-fold error on average, demonstrating that these Vinh sera were *at least* 20-fold degenerate.^25^ This corroborates recent work showing that different individuals often target the same epitopes,^26^ which should limit the number of distinct functional behaviors. As studies continue to measure sera in new locations, their transferability will quantify the level of heterogeneity across the world.

To demonstrate the scope of new antibody-virus interactions that can be inferred using available data, we predicted 2,000,000 new interactions between the Fonville and Vinh sera and their combined 81 H3N2 viruses. Upon stratifying by age, these landscapes can quantify how different exposure histories shape the subsequent antibody response.^25^^,^^39^ Given the growing interest in universal influenza vaccines that inhibit diverse variants, these high-resolution responses can examine the breadth of the antibody response both forwards in time against newly emerging variants and backwards in time to assess how rapidly immunity decays.^3^^,^^23^^,^^40^^,^^41^ We found that serum potency (the minimum HAI titer against a set of viruses) decreases for more distinct viruses (Figure 7B), as shown for monoclonal antibodies,^7^^,^^29^ suggesting that there is a tug-of-war between antibody potency and breadth. For example, a specific HAI target (e.g., responses with HAI ≥ 80 against multiple variants) may only be possible for viruses spanning 1–2 decades.

Our framework inspires new principles of data acquisition, where future studies can save time and effort by choosing smaller virus panels that are designed to be subsequently expanded (Figure 6A). One powerful approach is to perform experiments in waves. A study measuring serum inhibition against 100 viruses could start by measuring 5 of these viruses that are widely spaced out in time. With these initial measurements, we can compute the values ± errors of the remaining viruses as well as the next 5 maximally informative viruses, whose measurements will further decrease prediction error. Each additional wave of measurements serves as a test for the predictions, and experiments can stop oncewhen enough measurements match the predictions.

Antibody-virus interactions underpin diverse efforts, from virus surveillance^4^ to characterizing the composition of antibodies within serum^20^^,^^30^^,^^42^^,^^43^ to predicting future antibody-virus coevolution.^44^^,^^45^ Although we focused on influenza HAI data, our approach can readily generalize to other inherently low-dimensional datasets, both in and out of immunology. In the context of antibody-virus interactions, this approach not only massively extends current datasets but also provides a level playing field where antibody responses from different studies can be directly compared using the same set of viruses. This shift in perspective expands the scope and utility of each measurement, enabling future studies to always build on top of previous results.

### Limitations of the study

For cross-study antibody-virus predictions, there must be partial overlap in either the antibodies or viruses used across datasets. We only investigated cases where the virus panels overlapped, and we found that studies should contain ≥5 viruses (whose data can inform one another’s inhibition) for accurate predictions. For example, pre-pandemic H1N1, post-pandemic H1N1, and H3N2 would all minimally inform one another and should be considered separately (or else both the estimated and actual prediction error will be large). While we mostly investigated influenza HAI data, further work should extend this analysis to other viruses, other assays, and even to non-biological systems. In each context, this framework combines datasets to predict the value ± uncertainty of unmeasured interactions, and it circumvents issues of reproducibility or low-quality data (i.e., garbage in, garbage out) by explicitly computing intra- and inter-study relationships in a data-driven manner.

## STAR★Methods

### Key resources table

**Table:** 

REAGENT or RESOURCE	SOURCE	IDENTIFIER
**Deposited data**		

Fonville influenza datasets	Fonville et al.^20^	https://doi.org/10.1126/science.1256427
Vinh influenza dataset	Vinh et al.^25^	https://doi.org/10.1038/s41467-021-26948-8

**Software and algorithms**		

Cross-study prediction algorithm	This paper	https://doi.org/10.5281/zenodo.8034507

### Resource availability

#### Lead contact

Further information and requests for resources and reagents should be directed to and will be fulfilled by the lead contact, Tal Einav (tal.einav@lji.org).

#### Materials availability

This study did not generate new materials.

### Method details

#### Datasets analyzed

Information about the Fonville and Vinh datasets (type of study, year conducted, and geographic location) is provided in Table S1. The number of sera, viruses, and missing measurements in each dataset is listed below the schematics in Figure 4. Every serum was unique, appearing in a single study. All Fonville viruses appeared in at least two studies (see Figure S7C for the distribution of viruses), enabling us to entirely remove a virus from one dataset and infer its behavior from another dataset.

Although the Vinh data contained H1N1 and H3N2 viruses, we only considered the H3N2 strains since this was the only subtype measured in the Fonville data. 4/6 of the Vinh viruses (H3N2 A/Wyoming/3/2003, A/Wisconsin/67/2005, A/Brisbane/10/2007, and A/Victoria/361/2011) were in the Fonville virus panels. We associated the remaining two viruses with the most similar Fonville strain based on HA sequence (Vinh virus A/Aichi/2/1968↔Fonville virus A/Bilthoven/16190/1968; Vinh virus A/Victoria/210/2009↔Fonville virus A/Hanoi/EL201/2009). While such substitutions may increase prediction error (which can be gauged through leave-one-out analysis), they also vastly increase the number of possible cross-study predictions.

#### Matrix completion on log_10_(HAI titers)

The hemagglutination inhibition (HAI) assay quantifies how potently an antibody or serum inhibits the ability of a virus to bind red blood cells. The value (or titer) for each antibody-virus pair corresponds to the maximum dilution at which an antibody inhibits this interaction, so that larger values represent a more potent antibody. This assay is traditionally done using a series of 2-fold dilutions, so that the HAI titers can equal 10, 20, 40…

As in previous studies, all analysis was done on log_10_(HAI titers) because experimental measurements span orders of magnitude, and taking the logarithm prevents biasing the predictions toward the largest values^7^ while also accounting for the declining marginal protection from increasing titers.^46^ Thus, when computing the distribution of errors (histogram in Figures 3, 4 and 5), each of *M*, *μ*, and *σ* are computed in log_10_. The only exception is that when presenting the numeric values of a prediction or its error, we did so in un-logged units so the value could be readily compared to experiments. An un-logged value is exponentiated by 10 (i.e., *σ*_Predict,log10_ = 0.3 for log_10_ titers corresponds to an error of *σ*_Predict_ = 10^0^^.3^ = 2-fold, with “fold” indicating an un-logged number). The following sections always refer to *M*, *μ*, and *σ* in log_10_ units.

In the Fonville dataset, we replaced lower or upper bounds by their next 2-fold increment (”<10”→5 and “≥1280”→2560). The Vinh dataset did not include any explicit bounded measurements, although their HAI titers were clipped to lie between 10 and 1810, as can be seen by plotting the values of any two viruses across all sera. Hence, the Vinh predictions in Figure 4 (Dataset_Infect,2_) contains multiple points on the left and right edges of the plot.

#### Error of the hemagglutination (HAI) assay

In the Fonville 2014 study, analysis of repeated HAI measurements showed that the inherent error of the assay is log-normally-distributed with standard deviation σ_Inherent_ ≈ 2-fold. This is shown by Figure S8B in Fonville et al.^20^ (neglecting the stack of not-determined measurements outside the dynamic range of the assay), where 40% of repeats had the same HAI value, 50% had a 2-fold discrepancy, and 10% had a 4-fold discrepancy.

#### Using decision trees to quantify the relationships between viruses

Decision trees are a simple, easily-interpretable, and well-studied form of machine learning. An advantage of decision trees is that they are fast to train and have out-of-the-box implementations in many programming languages. The predictions from decision trees are made even more robust by averaging over the 5 top trees to create a small random forest, and we use such a “random copse” in this work. Similar approaches averaging across multiple decision trees (as well as variations such as survival decision trees) have been applied in various biological settings including genomics data and cancer.^47^^,^^48^

As described in Box 1, we trained regression trees that take as input the row-centered log_10_(HAI titers) from viruses *V*_1_-*V*_5_ to predict another virus *V*_0_. These trees can then be applied in another dataset to predict *V*_0_ based on the values of *V*_1_-*V*_5_.

Row-centering means that if we denote the log_10_(titers) of *V*_0_-*V*_5_ to be *t*_0_-*t*_5_ with mean *t*_avg_, then the decision tree will take (*t*_1_-*t*_avg_, *t*_2_-*t*_avg_, *t*_3_-*t*_avg_, *t*_4_-*t*_avg_, *t*_5_-*t*_avg_) as input to predict *t*_0_-*t*_avg_. The value *t*_avg_ (which will be different for each serum) is then added to this prediction to undo the row-centering. If any of the *t*_*j*_ are missing (including *t*_0_ when we withhold *V*_0_’s values), we proceed in the same way but compute *t*_avg_ as the average of the measured values. Row-centering enables the algorithm to handle systematic differences in data, including changes to the unit of measurement; for example, neutralization measurements in μg/mL or Molar would both be handled the same, since in log_10_ they are offset from each other by a constant factor that will be subtracted during row-centering. If one serum is concentrated by 2x, its titers would all increase by 2x but the relationships between viruses would remain the same; row-centering subtracts this extra concentration factor and yields the same analysis.

We chose a random fraction *f*_Samples_ = 3/10 of sera to train each decision tree when HAI data was continuous (Dataset_Infect,2_). For the remaining datasets with discrete measurements, we grouped sera based on the HAI titer of their virus-of-interest *V*_0_ (either HAI = 5, 10, 20, 40, 80, 160, or ≥320), picked among these bins with uniform probability, and then randomly chose a serum within that bin. This prevents the uneven HAI distribution from overwhelming the model, since the majority of measurements are HAI = 5 with very few cases of HAI≥320. This form of sampling minimally affected most predictions, but it improved the estimated error for human→ferret predictions (*σ*_Predict_ = 4.2x with this binning, *σ*_Predict_ = 6.4x with completely uniform binning), since HAI values in the ferret dataset within the limit of detection are not skewed toward low titers.

When training our decision trees, we allow missing values for *V*_1_-*V*_5_ but not *V*_0_ (as shown by the schematic in Figure 2B), with these missing values replaced by the most likely value (i.e., mode-finding) given the known values in the training set. When applying a trained decision tree to other datasets, we only predicted a value for *V*_0_ when none of *V*_1_-*V*_5_ were missing (otherwise that decision tree was ignored). If all 5 top trees were ignored due to missing values, then no prediction was made for that virus *V*_0_ and serum combination.

#### Predicting the behavior of a new virus

As described in Box 1, the values for *V*_0_ predicted from dataset *D*_*j*_→*D*_0_ is based on the top 5 decision trees that predict *V*_0_ in *D*_*j*_ with the lowest *σ*_Training_. The value of *V*_0_ against any serum is given by the average value of the top 5 decision trees, while its error is given by the estimated error *σ*_Predict_ = $f_{D_{j}\to D_{0}}$(*σ*_Training_) of these top 5 trees, where $f_{D_{j}\to D_{0}}$ represents the transferability map (described in the next section). Thus, every prediction of *V*_0_ in *D*_0_ will have the same *σ*_Predict_, unless some of the top 5 trees cannot cast a vote because their required input titers are missing (in which case the value ± error is computed using the average from the trees that can vote). In practice, the estimated error for *V*_0_ in *D*_0_ is overwhelmingly the same across all sera, as seen in Figure 3 where the individual error of each measurement is shown via error bars.

In Figures 4 and 5, we did not display the small fraction of measurements with HAI titers≥640 to better show the portions of the plots with the most points. However, the estimated error *σ*_Predict_ and true error *σ*_Actual_ were computed using all data.

#### Transferability maps between datasets

Transferability quantifies how the error of a decision tree trained in dataset *D*_*j*_ translates into this tree’s error in dataset *D*_0_. Importantly, when predicting the behavior of a virus *V*_0_ in *D*_0_, we *cannot* access *V*_0_’s values and hence cannot directly compute the actual error of this tree.

To solve this problem, we temporarily ignore *V*_0_ and apply Box 1 to predict the titers of viruses measured in both *D*_0_ and *D*_*j*_. Using the values of these viruses from both datasets, we can directly compare their *σ*_Training_ in *D*_*j*_ against *σ*_Actual_ in *D*_0_. We did not know *a priori* what the relationship would be between these two quantities, yet surprisingly, it turned out to be well-characterized by a simple linear relationship $f_{D_{j}\to D_{0}}$ (blue lines in Figure S1; if curves fall below the diagonal line, they are set to *y* = *x* since cross-study error should never fall below within-study error). As described in the following paragraph, these relations represent an upper bound, *not* a best fit, through the (*σ*_Training_, *σ*_Actual_) points, so that our estimated error *σ*_Predict_ ≡ $f_{D_{j}\to D_{0}}$ (*σ*_Training_) ≥ *σ*_Actual_. Therefore, when we estimate a small *σ*_Predict_ we expect *σ*_Actual_ to be small; a large *σ*_Predict_ may imply a large *σ*_Actual_, although we may also be pleasantly surprised with a smaller actual error.

Following Box 2, we obtain the best-fit line to these data using perpendicular offsets, which are more appropriate when we expect equal error in the *x*- and *y*-coordinates. To account for the scatter about this best-fit line, we add a vertical shift given by the RMSE of the deviations from the best-fit line, thereby ensuring that in highly-variable cases where some trees have small *σ*_Training_ but large *σ*_Actual_ (e.g., Dataset_Ferret_→Dataset_Vac,1_), we tend to overestimate rather than underestimate the error.

To visualize the transferability maps between every pair of datasets, we construct a chord diagram where the arc connecting Dataset *X* and *Y* represents a double-sided arrow quantifying both the transferability from *X*→*Y* (thickness of the arc at Dataset *Y*) as well as the transferability from *Y*→*X* (thickness of the arc at Dataset *X*) (Figure S4). The width of each arc is equal to Δ*θ*≡(2*π*/18.5)(*∂*$f_{D_{j}\to D_{0}}$/*∂σ*_Training_)^−1^, so that the width is proportional to 1/slope of the transferability best-fit line from Figure S1. We used the factor 18.5 in the denominator so that the chord diagrams in Figures 4B and 5B would form nearly complete circles, and if more studies are added this denominator can be modified (increasing it would shrink all the arcs proportionally). Note that the size of the arcs in Figures 4B and 5B can be directly compared to one another, so that if the arc from *X*→*Y* is wider in one figure, it implies more transferability between these datasets. A chord connects every pair of studies, unless there were fewer than 5 overlapping viruses between the studies (e.g., between Dataset_Infect,2_ and Dataset_Vac,3/4_), in which case the transferability could not be computed.

The transferability in Figures 4B and S1 represents all antibody-virus data, which is slightly different from the transferability maps we use when predicting virus *V*_0_ in dataset *D*_0_. When withholding a virus, we made sure to remove all trees from Figure S1 that use this virus as a feature. Although this can slightly change the best-fit line, in practice the difference is minor. However, when withholding multiple viruses in our leave-multi-out analysis, the number of datapoints in Figure S1 substantially decreased, and to counter this we trained additional decision trees (as described in the following section).

#### Leave-multi-out analysis

To withhold multiple viruses, we trained many decision trees using different choices of viruses *V*_1_-*V*_5_ to predict *V*_0_ in different datasets. Note that for leave-one-out analysis, we created 50,000 trees which provided ample relationships between the variants. However, when we withheld 133 viruses during the leave-multi-out analysis, we were careful to not only exclude decision trees predicting one of these withheld viruses (as *V*_0_), but to also exclude decision trees using any withheld virus in the feature set (in *V*_1_-*V*_5_). As a result, only 6,000 trees out of our original forest remained, and this smaller number of trees leads to higher *σ*_Predict_ and *σ*_Actual_ error. Fortunately, this problem is easily countered by growing additional trees that specifically avoid the withheld viruses. Once these extra trees were grown, we applied Box 1 as before.

To find a minimal virus panel, we randomly choose one of the 317 virus-study pairs from the Fonville/Vinh datasets, adding it to the list of withheld viruses provided that all withheld entries could be predicted with ≤4-fold error. We note that given a forest of decision trees, it is extremely fast to test whether a set of viruses all have *σ*_Predict_ ≤ 4-fold. However, as described above, as more viruses are withheld, our forest is trimmed which leads to poorer estimations of *σ*_Predict_. Hence we worked in stages, interspersing pruning the list of viruses with growing more decision trees. Our procedure to find a minimal virus panel proceeded in three steps.

• **Step 1: Choose Vinh viruses to withhold, and then choose viruses from the Fonville human studies.** Because there are only 6 Vinh viruses, and removing any one of them from the Fonville datasets could preclude making any Vinh predictions, we first withheld 2 Vinh viruses. We then started withholding viruses from the Fonville human datasets (Dataset_Vac,1-4_ and Dataset_Infect,1_) where we had the most decision trees.
• **Step 2: Create an additional random forest for the Fonville ferret dataset (Dataset**_**Ferret**_**).** This forest only used the non-withheld viruses from the other datasets as features. With this forest, choose additional viruses from the ferret dataset to withhold.
• **Step 3: Create additional random forests for the Fonville human datasets.** Use the improved resolution provided by these new forests to determine if any of the previously withheld viruses now have *σ*_Predict_ > 4-fold and remove them. Finally, use the additional high-resolution forests to search for additional Fonville viruses to withhold.

#### Extending virus panels

To extend the Fonville and Vinh datasets, we grew another forest of decision trees. Unlike in our leave-one-out analysis, the two key differences with this forest were that none of the data were withheld and that the feature set when expanding dataset *D*_0_ was restricted to only the viruses within *D*_0_. For example, to expand the Vinh dataset and predict one of the 81-6 = 75 Fonville viruses *V*_0_ (excluding the 6 viruses already in the Vinh data), we only searched for relationships between the six Vinh viruses and *V*_0_ across the Fonville datasets.

After growing these additional trees, we predicted the behavior of all 81 Fonville viruses against nearly every serum analyzed in the Fonville or Vinh datasets. The exceptions were sera such as those shown in the middle and bottom of Datasets_Vac,1/2_ (Figure S7) ‒ these sera were measured against few viruses, and hence we found no relationship between their available measurements in our random forest. The expanded virus panels are available in the GitHub repository associated with this paper.

With the expanded panels, we computed the tradeoff between serum potency and breadth as follows. For every range of Δvirus years, we considered every interval within our dataset (1968–1970, 1969–1971, …, 2009–2011), provided that at least one virus in the panel circulated at the earliest and latest year to ensure that the virus set spanned this full range (e.g., we would not consider the interval 1971–1973 since we had no viruses from 1971 or 1973). For each interval, we took whichever of the 81 viruses circulated during that interval, and for each serum we computed the weakest response (minimum titer) against any virus in this set. Figure 7B plots the largest minimum titer (HAI_min_) found in each dataset for any interval of Δvirus years, demonstrating that serum potency decreases when inhibiting viruses spanning a broader range of time.

### Quantification and statistical analysis

Details on the statistical details can be found in the figure captions and the method details section above. Errors (*σ*) were calculated as the root-mean-squared error of log_10_(titers), which were then exponentiated by 10 to un-log the result. All analysis was carried out in Mathematica.

## Data Availability

• Source data statement: This paper analyzes existing, publicly available data. The accession numbers for the datasets are listed in the key resources table. • Code statement: All original code has been deposited in GitHub (https://github.com/TalEinav/CrossStudyCompletion) and is publicly available as of the date of publication. The DOI is listed in the key resources table. This repository includes code to perform matrix completion in *Mathematica* and *R*, as well as the expanded Fonville and Vinh datasets shown in Figure S7. • Any additional information required to reanalyze the data reported in this paper is available from the lead contact upon request.

## Supplemental information

Document S1. Figures S1–S10 and Table S1

Document S2. Article plus supplemental information
