# Supplementary material for: Using interpretable machine learning to extend heterogeneous antibody-virus datasets
Source: Cell Rep Methods. 2023 Jul 25;3(8):100540. doi: 10.1016/j.crmeth.2023.100540 (PMC10475791; doi:10.1016/j.crmeth.2023.100540)
Supplement: Document S1. Figures S1–S10 and Table S1 [file mmc1.pdf]

Cell Reports Methods, Volume *3*

Supplemental information

Using interpretable machine learning to extend heterogeneous antibody-virus datasets

Tal Einav and Rong Ma

Supplemental Figures and Tables

Influenza Dataset	Organism	Type	Year Conducted	Geographic Location	Source of Data
$\text{Dataset}_{\text{Vac},1}$	Human	Vaccination	1997	Parkville, Australia	Fonville 2014, Table S5
$\text{Dataset}_{\text{Vac},2}$	Human	Vaccination	1998	Parkville, Australia	Fonville 2014, Table S6
$\text{Dataset}_{\text{Vac},3}$	Human	Vaccination	2009	Parkville, Australia	Fonville 2014, Table S13
$\text{Dataset}_{\text{Vac},4}$	Human	Vaccination	2010	Parkville, Australia	Fonville 2014, Table S14
$\text{Dataset}_{\text{Infect},1}$[(b)]	Human	Infection	2007-2012	Ha Nam, Vietnam	Fonville 2014, Table S3
$\text{Dataset}_{\text{Infect},2}$[(b)]	Human	Infection	2009-2015	Ha Nam, Vietnam	Vinh 2021 Supplement
$\text{Dataset}_{\text{Ferret}}$[(a)]	Ferret	Infection	N/A	N/A	Fonville 2014, Table S1

Table S1. Datasets analyzed in this work, related to STAR Methods. Information about the type of study as well as the year and geographic location from which the antibody responses were collected.

[(a)] Infected influenza-naive ferrets with a single virus and measured their serum against a panel of viruses.

[(b)] Over multiple years, participants reported influenza-like illnesses and got PCR tested. Serum samples were collected from all participants once each year.

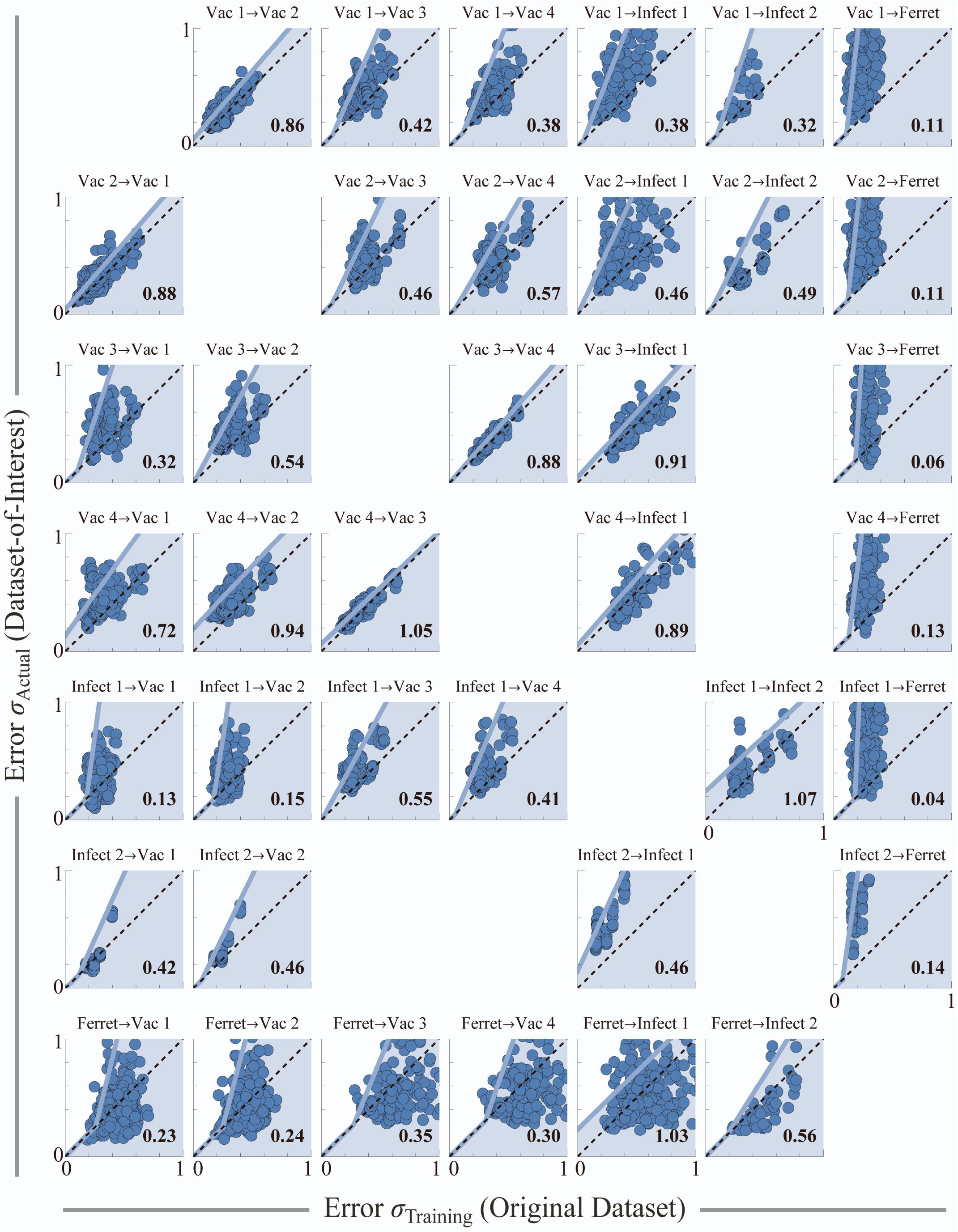

Figure S1. Transferability between datasets examined in this work, related to STAR Methods. Each plot quantifies the transferability relation $f_{j\to k}$ of virus behavior between Dataset j and Dataset k; the relation $f_{j\to k}$ represents an upper bound (*not* a best fit-line), with the majority of points expected to lie within the shaded region. Each point represents a decision tree trained on 30% of samples in Dataset j, with its cross-validation RMSE σ_{Training} computed on $\log_{10}$(titers) against the remaining 70% of samples [x-axis]. This tree was then applied to Dataset k, with RMSE σ_{Actual} [y-axis]. Every possible virus (measured in both Dataset j and Dataset k) was withheld and predicted, and the plotted points represent the 5 decision trees with the lowest σ_{Training} (or the top 10 trees if there are fewer than 300 points in the plot to ensure sufficient sampling). The best-fit perpendicular line $f_{\perp}$ was fit to the resulting points, and to account for variability (and to overestimate rather than underestimate error) we add to this line the constant f_{RMSE} (the RMSE of the vertical deviations between $f_{\perp}$ and each point). Lastly, because error should increase when extrapolating the predictions to a new dataset ($\sigma_{\text{Training}} \leq \sigma_{\text{Actual}}$), and because some of the lines are nearly vertical, we enforce that $f_{j\to k}$ lies above $y=x$ by defining $f_{j\to k}=\max(f_{\perp}+f_{\text{RMSE}}, \sigma_{\text{Training}})$. The only plots that are not shown are the diagonal entries (we do not need self-transferability) and Vac 3/4 and Infect 2 (these datasets only have 1 overlapping virus which is not enough to quantify transferability; hence no predictions were made between these datasets). The numbers at the bottom-right of each plot show the transferability, 1/(slope of $f_{\perp}+f_{\text{RMSE}}$).

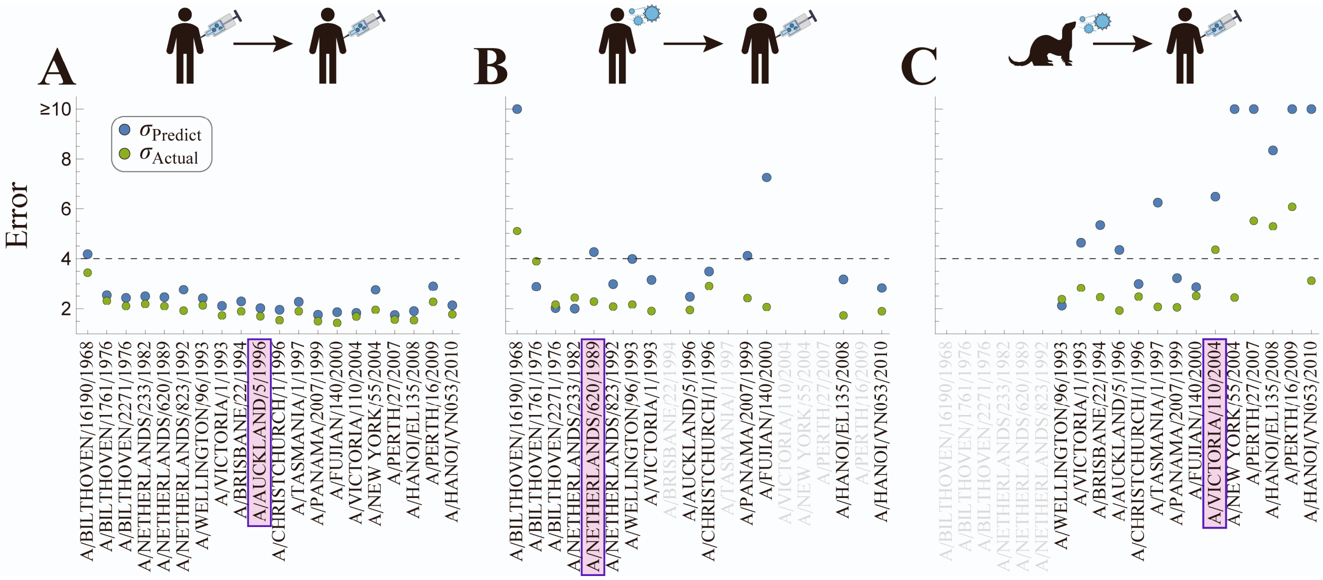

Figure S2. Predicting each virus in Dataset$_{Vac,4}$ using one other dataset, related to Figure 3. We withhold one virus in Dataset$_{Vac,4}$ (*x*-axis) and predict it using (A) the human vaccination study [Dataset$_{Vac,3}$], (B) the human infection study [Dataset$_{Infect,1}$], or (C) the ferret infection study (Dataset$_{Ferret}$). In each case, we show the estimated error ($\sigma_{Predict}$, blue) and the true error (σ_{Actual}, green). Viruses appear in the same order in each plot, sorted by year of circulation. Grayed-out viruses could not be predicted either because they were absent from a dataset (*e.g.*, Dataset$_{Infect,1}$ did not contain A/Brisbane/22/1994) or because of insufficient data. The three viruses shown in Figure 3 are boxed in purple. 1-fold error (bottom of plots) represents a perfect theory-experiment match; dashed line represents the 4-fold error point of reference used throughout this work.

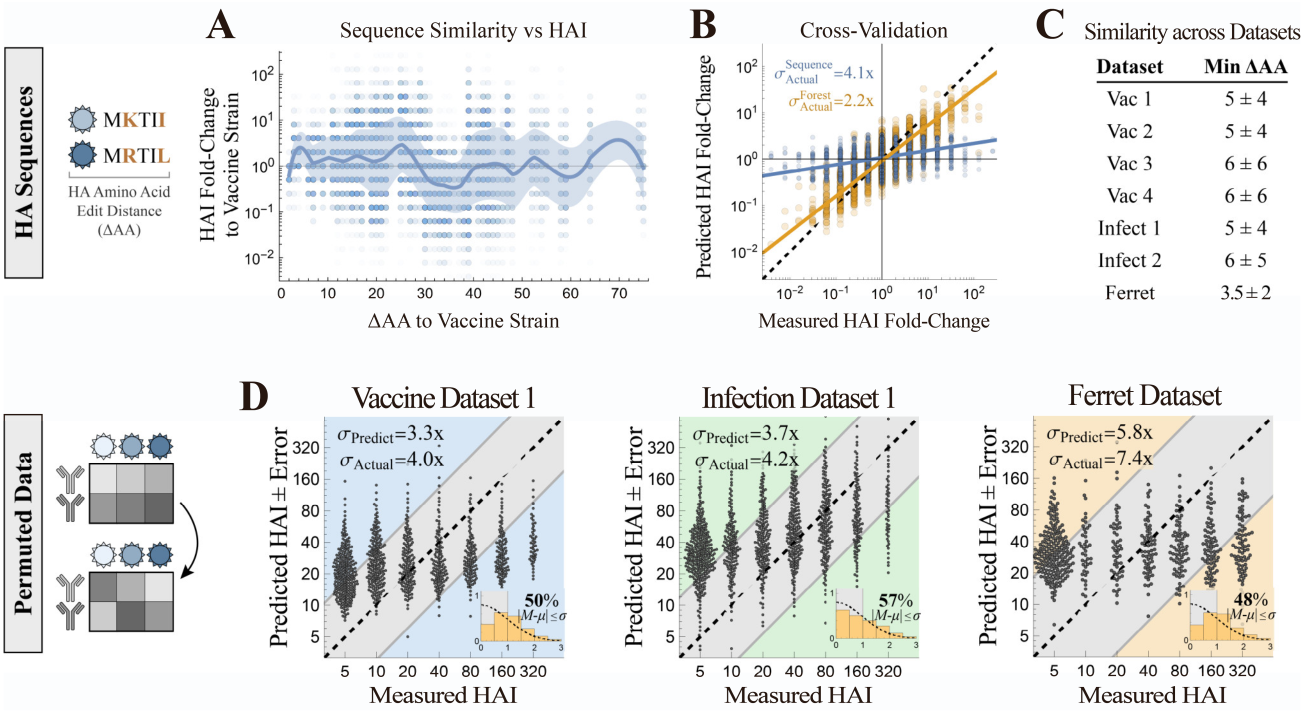

Figure S3. Quantifying the effects of sequence similarity and permutation analysis on HAI predictions, related to Figure 4. (A) Amino acid edit distance (ΔAA) between each virus' HA sequence and the vaccine strain HA sequence

plotted against the fold-change in HAI between the virus and vaccine strain. The solid line shows an interpolation of the mean±standard deviation. Analysis was performed for all sera in Datasets$_{\text{Vac},1\text{-}4}$ whose vaccine strains were H3N2 A/Nanchang/933/1995, A/Sydney/5/1997, A/Brisbane/10/2007 [substituted by the closest analogue A/Perth/27/2007 since the vaccine strain was not in the virus panel], and A/Perth/16/2009, respectively. (B) Cross-validation of this approach [blue points] using 30% of the sera to interpolate the relationship in Panel A and then predict the HAI of the remaining sera, repeated 10 times to avoid sampling bias. Leave-one-out predictions from Figure 4 are shown for comparison [gold points] as fold-change relative to the vaccine strain. (C) Quantifying the most similar viruses in leave-one-out analysis. For each virus-of-interest in dataset X, we take all datasets $\{Y_1, Y_2\ldots\}$ containing the virus-of-interest and find the smallest amino acid distance (min ΔAA) to the viruses those datasets (excluding the virus-of-interest). Statistics show the mean±standard deviation over all viruses in each dataset. (D) Permutation testing was performed by randomly permuting the measured titers in the Fonville datasets and performing leave-one-out analysis as in Figure 4A. Resulting predictions are shown for one vaccine, infection, and ferret dataset, each of which resulted in a larger σ_{Actual} than in the original analysis in Figure 4A (unpermuted data available in GitHub repository).

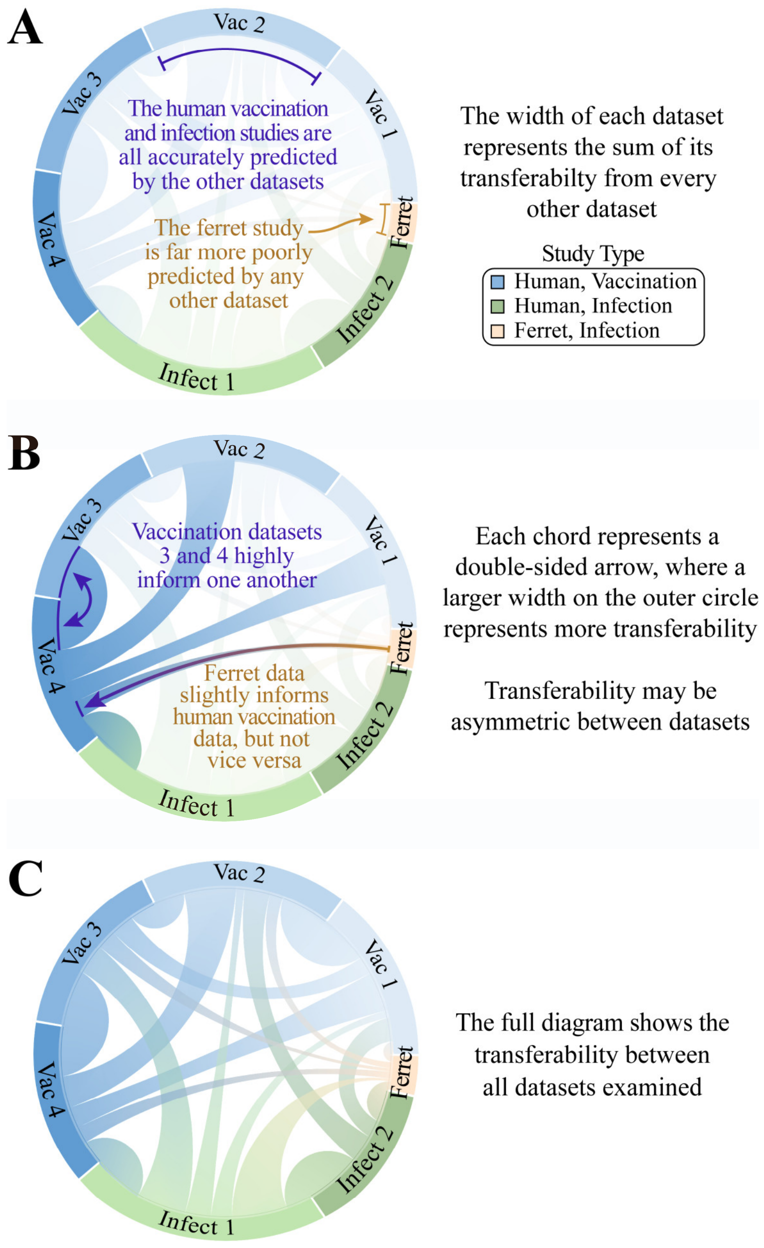

Figure S4. Explaining the chord diagram, related to Figure 4. The chord diagram in Figure 4B represents the transferability between the influenza datasets when considering all data. (A) The width of each dataset represents the sum of its transferability from all other datasets. This total width is not directly used (we only use the transferability between each pair of studies), but the smaller total width of the ferret study indicates that all other datasets poorly infer the ferret measurements. (B) A wider arc from Study $X \rightarrow$ Study Y represents greater transferability. More precisely, transferability equals 1/slope of the linear map in Figure S1, so that studies with near-perfect transferability (slope$\approx$1) will have large width while studies with poor transferability (slope$\gg$1) will have small width. (C) The full diagram from Figure 4B.

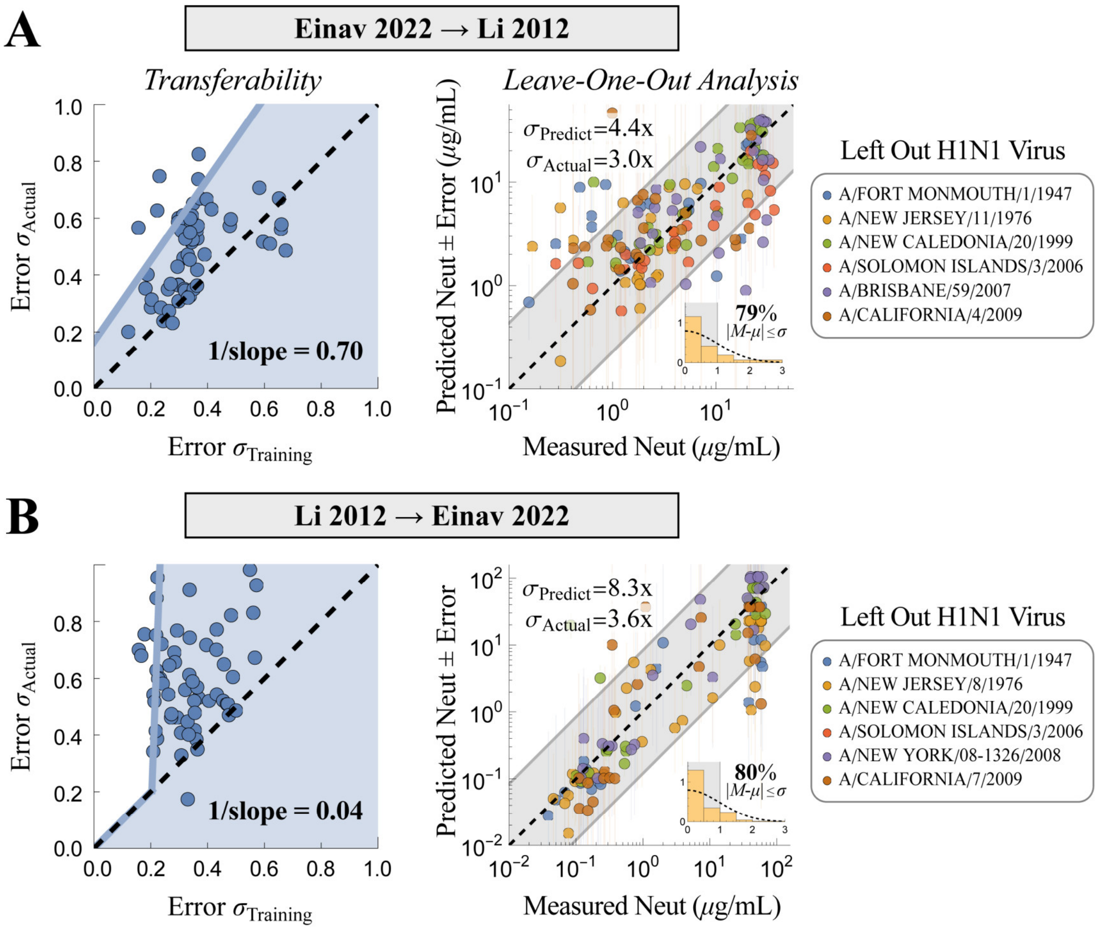

Figure S5. Predictions between two monoclonal antibody datasets measuring H1N1 virus neutralization, related to STAR Methods. As in the main text, we entirely removed one of the six viruses from (A) Li *et al*. (Li et al., 2012) and predicted its neutralization using data from Einav *et al*. (Einav et al., 2022) and (B) vice versa. *Left*, the transferability between datasets; *Right*, predictions for each withheld virus, with individual error shown on each point and average error shown by the gray diagonal band. Despite differences in the neutralization assay (IC_{100} [100% inhibitory concentration] in Li 2012 versus IC_{50} in Einav 2022), both datasets yield predictions with accuracy σ_{Actual}=3.0–3.6-fold. The higher transferability from Einav 2022→Li 2012 leads to a tighter upper bound σ_{Predict} on σ_{Actual}. For this analysis, we equated the nearly homologous strains A/New Jersey/8/1976≈A/New Jersey/11/1976 (ΔAA=0; amino acid edit distance calculated using consensus HA sequences from GISAID), A/California/4/2009≈A/California/7/2009 (ΔAA=1), and A/Brisbane/59/2007≈A/New York/08-1326/2008 (ΔAA=2).

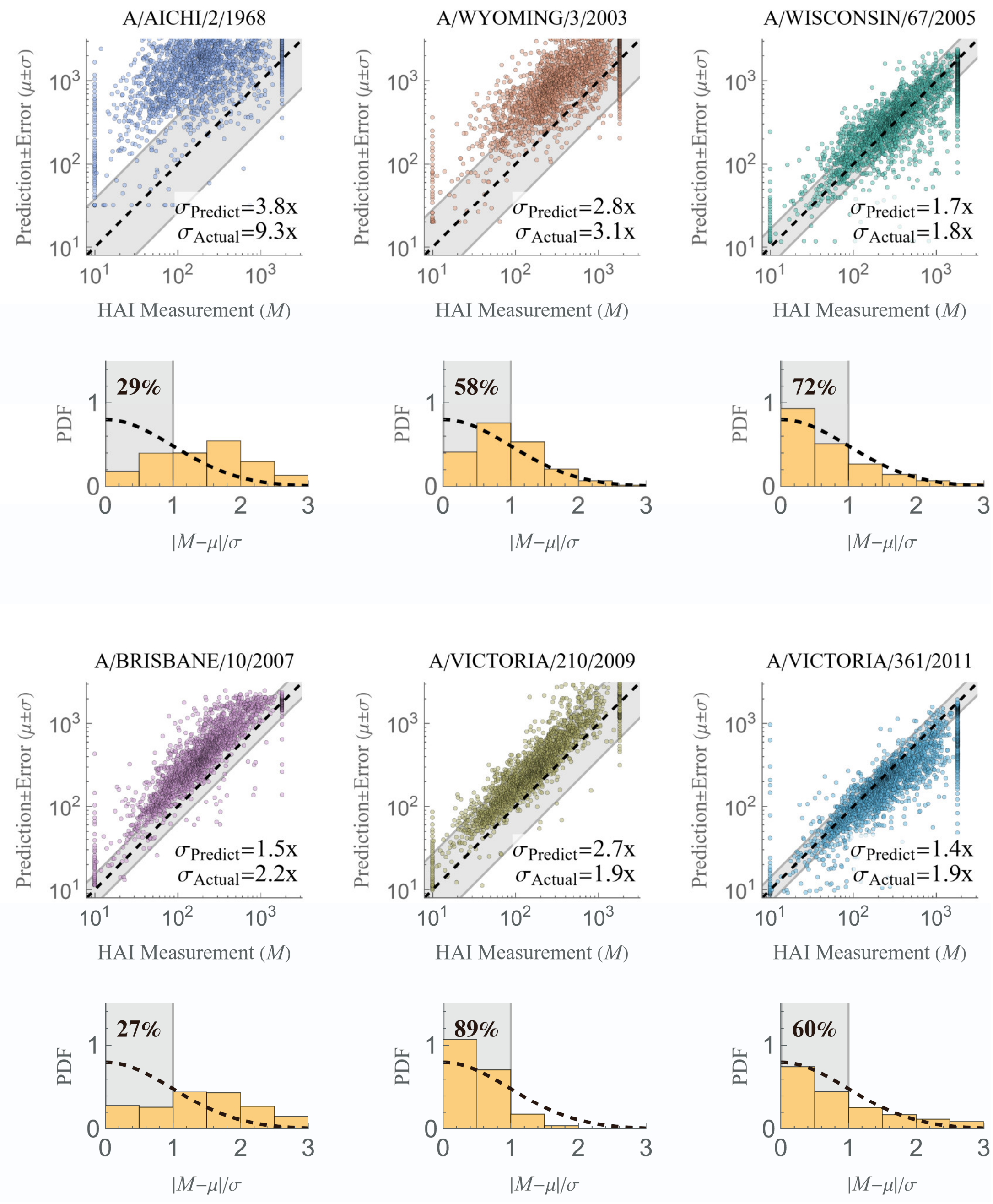

Figure S6. Individual matrix completions in the Vinh dataset, related to Figure 4. Each of the six Vinh viruses were withheld and predicted using the Fonville data. *Scatterplots* show predictions versus measurements. For each virus, the uncertainty of its predictions will be the same for all 25,000 values, and this uncertainty is visualized using the gray bands (showing the fold-error σ_{Predict}); the predicted and true errors are also written at the bottom-right of each plot. For clarity, we only show every 10th data point of the 25,000 measurements, but all statistics are computed using the full data. *Histograms* portray the error distribution for the predictions, with the value in the gray region showing the number of predictions within 1σ of the measurement.

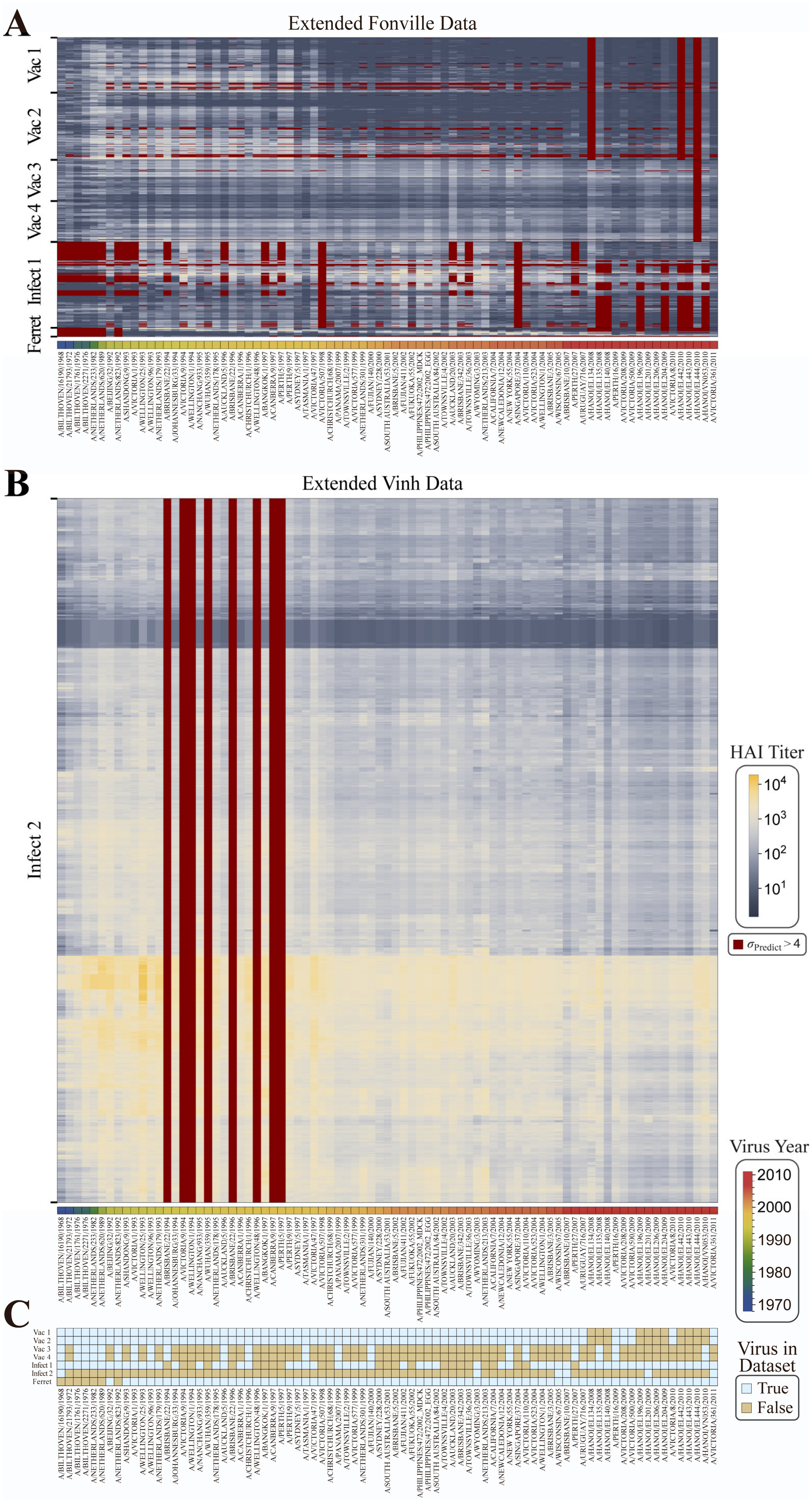
A
Extended Fonville Data
Vac 1
Vac 2
Vac 3
Vac 4
Infect 1
Ferret
B
Extended Vinh Data
Infect 2
HAI Titer
10^4
10^3
10^2
10^1
$\sigma_{Predict} > 4$
Virus Year
2010
2000
1990
1980
1970
C
Vac 1
Vac 2
Vac 3
Vac 4
Infect 1
Infect 2
Ferret
Virus in Dataset
True
False

Figure S7. Expanded HAI titers for all datasets considered in this work, related to Figure 6. Using the available measurements, we predicted all antibody-virus interactions in the (A) Fonville and (B) Vinh datasets. In total, we added 32,000 and 1,600,000 new measurements with ≤4-fold error in the Fonville and Vinh datasets, respectively; all other predictions with $\sigma_{Predict}>4$ are shown in dark red. The sera in each dataset were clustered based on their Ward similarity function. Viruses are ordered by their year of circulation in both plots, and the color in the bottom row represents a virus's year of circulation. The complete list of measurements and predictions is included in the associated GitHub repository. (C) Distribution of viruses across datasets. For cross-study comparison, two viruses in $Dataset_{Infect,2}$ were equated with their closest virus (A/Aichi/2/1968↔A/Bilthoven/16190/1968 and A/Victoria/210/2009↔A/Hanoi/EL201/2009, Methods).

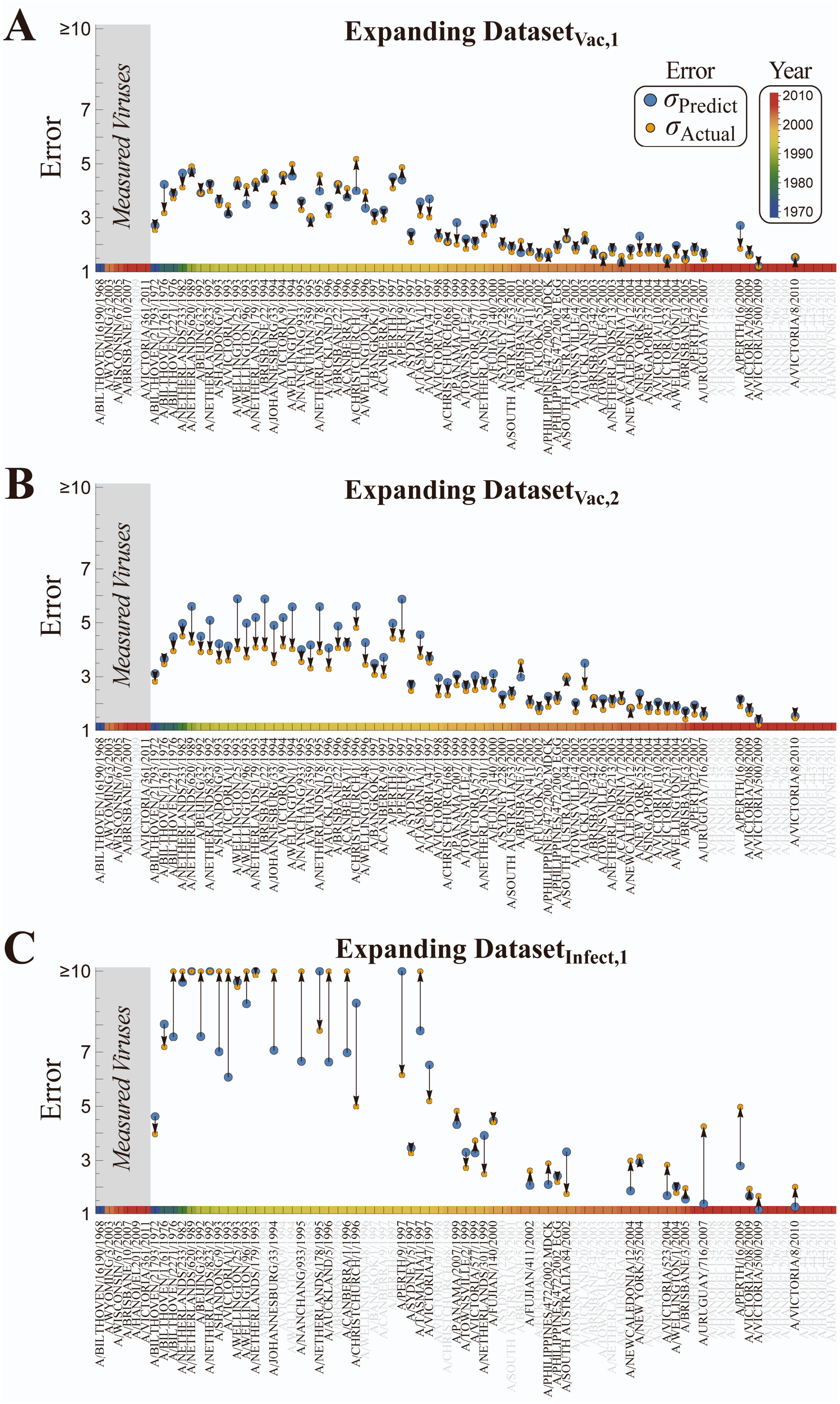

Figure S8. Extrapolating virus behavior in the Fonville datasets using 6 viruses, related to Figure 6. Analogous to Figure 6, we only use values from the six Vinh viruses (or the subset of these viruses present in each Fonville dataset) to

predict the behavior of all other viruses. We consider predictions in (A) Dataset$_{Vac,1}$, (B) Dataset$_{Vac,2}$, or (C) Dataset$_{Infect,1}$, which are the three datasets that contribute the most of the Vinh predictions [Figure 4B]. Each plot shows the predicted error [$\sigma_{Predict}$, blue] and actual error [σ_{Actual}, gold], with a connecting arrow. Viruses in gray could not be predicted either because they were not in the Fonville dataset or there was insufficient data. Viruses from the 1980s and 1990s (which are the furthest away from the 5-6 measured viruses) have the largest error, and this error is slightly overestimated in Dataset$_{Vac,2}$ and underestimated in Dataset$_{Infect,1}$. As explained in the Methods, our framework is constructed so that low $\sigma_{Predict}$ always implies a low σ_{Actual} (with $\sigma_{Predict}\approx\sigma_{Actual}$), whereas large $\sigma_{Predict}$ implies less certainty in σ_{Actual}. A good rule of thumb from these results is to not use values with a predicted error ≥6-fold, since their true error may be even larger; we note that all inferred values in Figure 6C have a predicted error <6-fold.

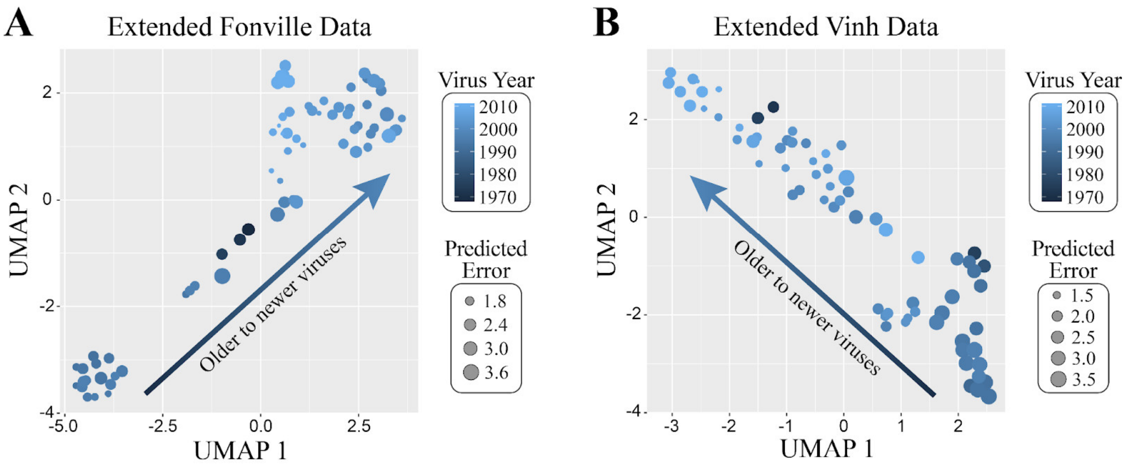

Figure S9. UMAP embeddings of the expanded antibody-virus datasets, related to Figure 6. We applied UMAP upon the expanded data from (A) Fonville and (B) Vinh, using the $\log_{10}$(HAI titers) with $n_{neighbor}=20$ and the default tuning parameters in the *R* package *uwot*. Each data point in the plot corresponds to a specific virus (81 in total), with the size of each point indicating the predicted error of the imputed values, and the shading indicating the year the virus circulated. In both UMAPs, the viruses show a clear temporal pattern moving along a straight line, even though this temporal information was never provided to the algorithm.

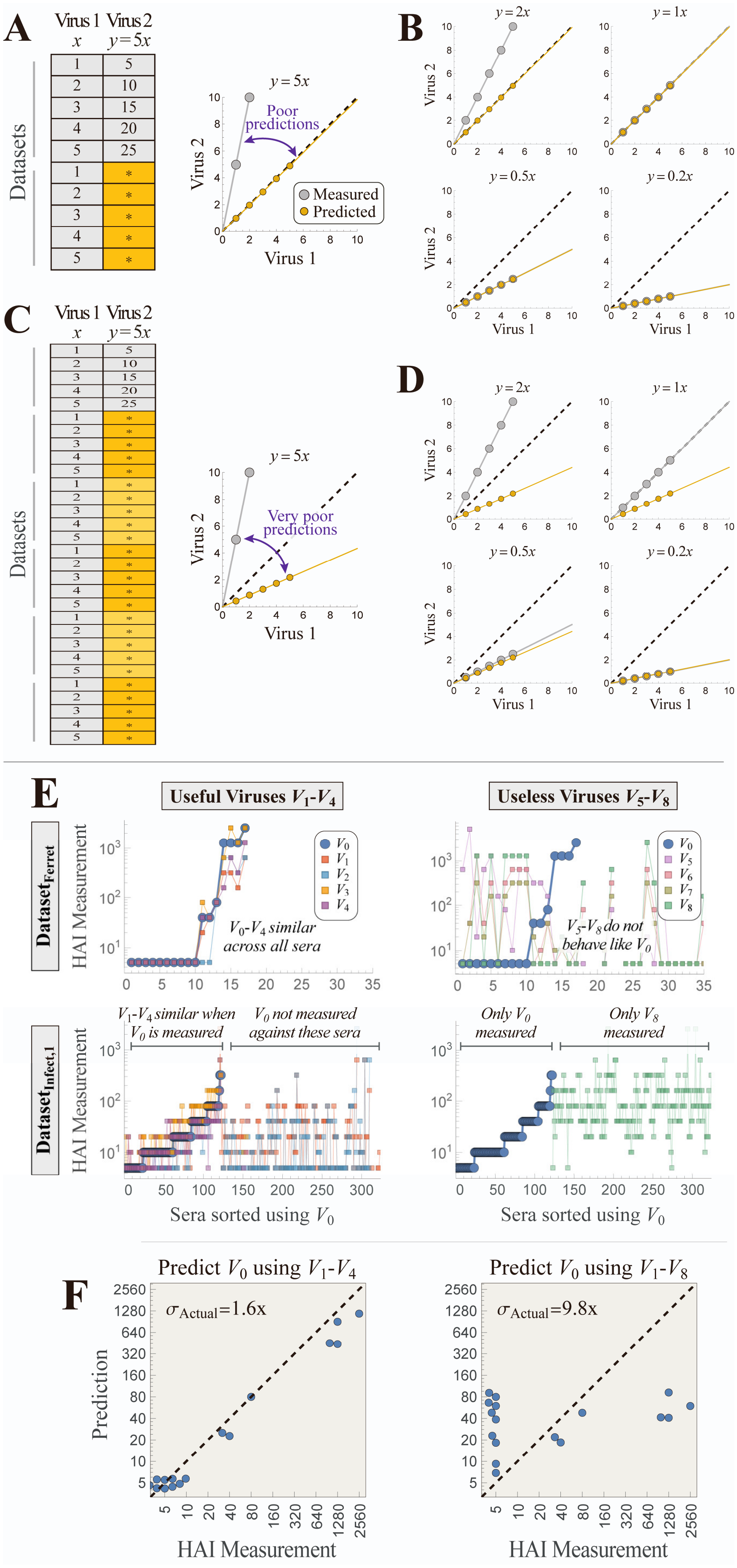

A
Virus 1
Virus 2
x
y = 5x
Datasets
y = 5x
Poor predictions
Measured
Predicted
Virus 2
Virus 1
B
y = 2x
y = 1x
y = 0.5x
y = 0.2x
Virus 2
Virus 1
C
Virus 1
Virus 2
x
y = 5x
Datasets
y = 5x
Very poor predictions
Virus 2
Virus 1
D
y = 2x
y = 1x
y = 0.5x
y = 0.2x
Virus 2
Virus 1
E
Useful Viruses V_1-V_4
Useless Viruses V_5-V_8
$\text{Dataset}_{\text{Ferret}}$
HAI Measurement
V_0-V_4 similar across all sera
V_5-V_8 do not behave like V_0
$\text{Dataset}_{\text{Infect,1}}$
V_1-V_4 similar when V_0 is measured
V_0 not measured against these sera
Only V_0 measured
Only V_8 measured
Sera sorted using V_0
F
Predict V_0 using V_1-V_4
Predict V_0 using V_1-V_8
σ_{Actual}=1.6x
σ_{Actual}=9.8x
Prediction
HAI Measurement

Figure S10. Artifacts of nuclear norm minimization (NNM) can lead to poor predictions, related to Figure 5. (A-D) NNM can fail in a simple, noise-free setting. (A) Toy example where measurements from two viruses are proportional ($y=5x$) and the input dataset has a perfect template of this relationship, but Virus 2 is incorrectly predicted as $y=x$. (B) This problem holds for any relation $y=mx$ where $m>1$, although values of $m\leq1$ lead to perfect recovery. (C,D) The problem is exacerbated when there are n copies of the missing measurements, with Virus 2 predicted as $y=n^{-1/2}x$ whenever $m>n^{-1/2}$. (E-F) NNM may give poor predictions when there are large swaths of missing values. Predictions for virus V_0 [specified below] from $\text{Datasets}_{\text{Infect},1\rightarrow\text{Ferret}}$ are highly accurate when using the "useful" viruses V_1-V_4 that behave similarly in both studies, but highly inaccurate when adding the additional "useless" viruses V_5-V_8 that don't behave like V_0 in either study. (E) Plot of the titers of the useful and useless viruses in both datasets, with sera sorted according to the HAI titers of V_0. Values for V_1-V_4 closely match those of V_0 for all sera in $\text{Dataset}_{\text{Ferret}}$ and for all sera where V_0 is measured in $\text{Dataset}_{\text{Infect},1}$ (the first 125 sera). In contrast, V_5-V_8 do not behave like V_0 in $\text{Dataset}_{\text{Ferret}}$; in $\text{Dataset}_{\text{Infect},1}$ viruses V_5-V_7 are never measured, and V_8 is only measured against sera where V_0 was not measured. Hence, V_5-V_8 should ideally not influence the matrix completion of V_0. (F) The resulting predictions vs measurements for V_0 only using V_1-V_4 [left] or using both V_1-V_4 and V_5-V_8 [right], with the latter leading to significantly larger error. In the Fonville datasets, these viruses represent V_0=VN018/EL204/2009, V_1-V_4={HN201/2009, HN206/2009, VN019/EL442/2010, VN020/EL443/2010}, and V_5-V_8={A/Singapore/37/2004, A/South Australia/53/2001, A/Sydney/228/2000, A/South Australia/84/2002}.
